# Tdp-25 Routing to Autophagy and Proteasome Ameliorates its Aggregation in Amyotrophic Lateral Sclerosis Target Cells

**DOI:** 10.1038/s41598-018-29658-2

**Published:** 2018-08-17

**Authors:** Maria Elena Cicardi, Riccardo Cristofani, Paola Rusmini, Marco Meroni, Veronica Ferrari, Giulia Vezzoli, Barbara Tedesco, Margherita Piccolella, Elio Messi, Mariarita Galbiati, Alessandra Boncoraglio, Serena Carra, Valeria Crippa, Angelo Poletti

**Affiliations:** 1 Department of Excellence: Dipartimento di Scienze Farmacologiche e Biomolecolari (DiSFeB), Centro di Eccellenza sulle Malattie Neurodegenerative, Università degli Studi di Milano, Milano, Italy; 2 Dipartimento di Scienze Biomediche, Metaboliche e Neuroscienze, Università degli Studi di Modena e Reggio Emilia, and Center for Neuroscience and Neurotechnology, Modena, Italy; 3 Centro InterUniversitario sulle Malattie Neurodegenerative, Università degli Studi di Firenze, Genova, Roma Tor Vergata and Milano Italy

## Abstract

Amyotrophic lateral sclerosis (ALS) is a fatal neurodegenerative disease that primarily affects motoneurons, while non-neuronal cells may contribute to disease onset and progression. Most ALS cases are characterized by the mislocalization and aggregation of the TAR DNA binding protein 43 (TDP-43) in affected cells. TDP-43 aggregates contain C-terminal TDP-43 fragments of 35 kDa (TDP-35) and 25 kDa (TDP-25) and have been mainly studied in motoneurons, while little is currently known about their rate of accumulation and clearance in myoblasts. Here, we performed a comparative study in immortalized motoneuronal like (NSC34; i-motoneurons) cells and stabilized myoblasts (C2C12; s-myoblasts) to evaluate if these two cell types differentially accumulate and clear TDP forms. The most aggregating specie in i-motoneurons is the TDP-25 fragment, mainly constituted by the “prion-like” domain of TDP-43. To a lower extent, TDP-25 also aggregates in s-myoblasts. In both cell types, all TDP species are cleared by proteasome, but TDP-25 impairs autophagy. Interestingly, the routing of TDP-25 fragment to proteasome, by overexpressing BAG1, or to autophagy, by overexpressing HSPB8 or BAG3 decreased its accumulation in both cell types. These results demonstrate that promoting the chaperone-assisted clearance of ALS-linked proteins is beneficial not only in motoneurons but also in myoblasts.

## Introduction

Amyotrophic lateral sclerosis (ALS) is a devastating adult onset neurodegenerative disease in which upper cortical and lower spinal cord motoneurons are primarily affected^1,2^. Only a low number of ALS cases (about 10%) occurs in an inherited form (familial ALS, fALS), while the higher number is represented by sporadic cases (sALS)^2^. The various fALS cases have been associated to mutations in different genes. The *C9ORF72* and the superoxide dismutase 1 (*SOD1*) genes are causative of more than 60% of all fALS. Other genes have been found mutated in rare fALS cases (e.g.: the *TARDBP* encoding the TAR DNA binding protein TDP-43, *OPTN1* encoding optineurin-1, *FUS* encoding fused in sarcoma, *UBQLN-2* encoding ubiquilin-2, etc.); surprisingly enough, even if not mutated, some of the protein products of these genes display an aberrant behavior as wild-type (WT) forms also in most sALS^3^. This suggests the existence of common mechanisms of disease in fALS and sALS. One of the best examples is represented by the protein TDP-43. In normal individuals, TDP-43 localizes to the nucleus, where it is involved in RNA metabolism^3^. Conversely, in almost all sALS cases TDP-43 mislocalizes into the cytoplasm of neuronal cells in the brain and spinal cord of the affected regions, where it aggregates^4–8^. TDP-43 accumulation is not restricted to ALS, but it may occur for example also in Frontolateral Temporal Dementia (FLTD)^6^ and in sporadic inclusion body myositis^9^. In ALS, neuronal TDP-43 is cleaved generating fragments of 35 and 25 kDa that are highly aggregation-prone, and exert neurotoxicity with unclear mechanisms. Sequestration by aggregating TDP fragments of wild type full-length (FL) TDP-43 protein, has been proposed to contribute to their toxicity leading to a loss of TDP-43 functions^10^. Therefore, TDP-35 or TDP-25 fragments must be efficiently cleared from cells to prevent their aggregation and sequestration of other important neuronal components, with toxic consequences^11^.

In mammalian cells, the clearance of aberrantly folded or misfolded proteins is mediated by the intracellular protein quality control (PQC) system^11^. The PQC system is composed of chaperone/co-chaperone proteins, which recognize, bind to and target aberrant proteins to degradation, and the degradative systems, like the ubiquitin proteasome system and the autophagy^12–14^. Chaperones and co-chaperones work in complex; one example is represented by BAG3, a co-chaperone of HSP70 that also binds to the chaperone HSPB8, and the E3 ligase CHIP. This complex is referred to as the CASA complex and targets misfolded proteins to autophagy^11,13,14^.

In mice models of ALS, these proteins are differentially up-regulated both in the spinal cord and in muscle cells^15–19^. In particular, muscle cells are reactive to the presence of misfolded species, such as mutant SOD1. In fact, the expression of PQC system proteins (e.g. HSPB8, BAG3, BAG1), along with markers of autophagy (SQSTM1 and LC3) is highly increased in muscle of tg ALS mice^17,18^, generally at higher levels than those found in the spinal cord of the same mice. However, the spinal cord is a multicellular tissue in which the relative contribution on potentially affected motoneuronal cells is much lower than that of other neurons, astrocytes or microglial cells. By directly comparing mutant SOD1 biochemical behavior in motoneuronal and muscle ALS cell models we previously established that muscle cells are characterized by a higher PQC activity compared to motoneuronal cells. Despite the higher capability of muscle than spinal cord to handle misfolded proteins, also the muscle has been described to be directly affected in ALS^20–24^.

Few data are available on the role of TDP-43, and its disease associated fragments in muscle cells. Some indications of TDP-43 involvement in muscle system arise from ALS zebrafish model recapitulating a loss-of-function of TDP-43. In animals exposed to selective antisense morpholino oligonucleotide to silence TDP-43 there was an increased number of orphaned pre- and postsynaptic neuromuscular junction (NMJ) markers^25^. In addition, a three to four folds increase of acetylcholine release was measured suggesting the importance of TDP-43 to prevent synaptic dysfunction in ALS^25^. How this relates to a specific role of TDP-43 in muscle in preventing NMJ loss is still unclear. However, a contribution of a loss of TDP-43 function, related to its nuclear depletion in affected cells, has been suggested by several investigators (see^26^ for review). Also, the over-expression of TDP-43 in mice leads to skeletal muscle alterations with a dysregulation of the Glucose 4 transporter (Glut4) and reduced insulin functions^27^. Muscle of ALS patients has a reduced glycolytic activity and a special need of fatty acids metabolism^28^. A recent study showed the existence of TDP-43 inclusions specific muscles (axial skeletal muscles, paraspinus and diaphragm) of some selected familial and sporadic ALS patients^29^. Unfortunately, so far no cell biological studies have been focused on the analysis of the biochemical behavior of TDP-43 and the ALS associated fragments in muscle.

In this study, we comparatively analysed how TDP-43 is processed and degraded in immortalized motoneuronal like (NSC34; i-motoneurons) cells and stabilized myoblasts (C2C12; s-myoblasts) cells. We found that the TDP-25 fragment is the mainly aggregating specie in i-motoneurons where it aggregates with a much higher rate than in s-myoblasts. We also found that i-motoneurons may be more affected than muscle cells by the presence of TDP-43 fragments because of their overall lower degradative power compared to s-myoblasts. We found that in both i-motoneurons and s-myoblasts, TDP-43 and fragments are cleared by the proteasome, but the TDP-25 fragments have a great impact on autophagy as shown by SQSTM1/p62 bodies formation in close association to the TDP-25 inclusions. By overexpressing a selective chaperone (HSPB8) and co-chaperones (BAG1 and BAG3) to modulate the degradative routing system, we found that both proteasome and autophagy efficiently removes TDP-25 inclusions in both cell types.

All data collected converge on the notion that muscle cells have a more efficient routing system between proteasome and autophagy than motoneuronal cells.

## Materials and Methods

### Chemicals

Z-Leu-Leu-Leu-al (MG132) was used at 10 µM for 16 hrs (Sigma-Aldrich, St. Loius, MO, USA). Bortezomib (Selleckem, Houston, TX, USA) was used at 200 nM for 16 hrs. Wortmaninn was used at 50 nM for 36 hrs (Sigma-Aldrich). DMSO was used as control (Sigma-Aldrich).

### Cell cultures

NSC34 cells were used as motor neuronal cell model. Cells were routinely used in our lab^11,13,30,31^. Briefly, cells were maintained in Dulbecco’s Modified Eagle Medium (DMEM) high glucose medium (Euroclone, Pero, MI, Italy) supplemented with 1 mM glutamine (Euroclone), penicillin (SERVA, Electrophoresis GmbH, Heidelberg, Germany) and streptomycin (SERVA) and 5% fetal bovine serum (Sigma-Aldrich). Cells were grown at 37 °C with 5% CO_2_. To differentiate NSC34, retinoic acid 1 μM (Sigma-Aldrich) was added to full medium for 24 hrs before transfection and maintained for 72 hrs. C2C12 cells were used as muscle model. Cells were cultured as previously described^32^, and maintained in DMEM high glucose medium completed with glutamine, penicillin, streptomycin and 10% of fetal bovine serum (GIBCO, Thermo Scientific Life Sciences Research, Waltham, MA, USA). Cells were grown at 37 °C with 5% CO_2_. C2C12 were differentiated by supplementing medium with 2% Horse Serum (HS; Sigma-Aldrich), instead of 10% FBS, for 72 hrs.

### Plasmids

The following plasmids were used in both cell lines: pEGFP-TDP-43, pEGFP-TDP-35 and pEGFP-TDP-25 which code for the TDP-43 variants fused with GFP protein at the N-terminus (kindly provided by Dr. Petrucelli); p2XFLAG-TDP-43, p2XFLAG-TDP-35, p2XFLAG-TDP-25 that code for the TDP-43 with the FLAG tag^11^; pDS-RED-monomer which codes for the monomeric DS-RED protein; pCI-hHSPB8 which codes for the human form of the small heat shock protein of 22 kDa, pCI-6xHis-FL-BAG3 which codes for the human form of full length BAG3, pcDNA/HA-Bag1, which codes for the human form of BAG1 (kindly provided by Dr. H.H. Kampinga). pcDNA3 by Invitrogen (Carlsband, CA, USA) was used as a transfection control.

### Transfection procedure

Undifferentiated and differentiated NSC34 cells were transfected using Lipofectamine® Transfection Reagent (Invitrogen, Thermo Scientific Life Sciences Research, Waltham, MA, USA)^32–34^. Plasmid DNA was firstly incubated with transferrin (Sigma-Aldrich)^35^ and then added with lipofectamine. Undifferentiated C2C12 cells were transfected with Lipofectamine® 2000 Transfection Reagent (Invitrogen) following manufacturer’ instructions. Differentiated C2C12 were transfected with Lipofectamine® 3000 Transfection Reagent (Invitrogen) following manufacturers’ instructions.

When experiments were carried out in 12-wells plates the following quantities of plasmids were used: 0.5 µg of pEGFP-TDP-43, pEGFP-TDP-35, pEGFP-TDP-25, p2XFLAG-TDP-43, p2XFLAG-TDP-35, p2XFLAG-TDP-25 and 0.6 µg of pDS-RED-monomer, pCI-hHSPB8, pCI-6xHis-FL-Bag3, pcDNA/HA-Bag1 or pcDNA3 as control. Plasmid quantities were halved or doubled respectively if experiments were carried out in 24-wells plate or in 6-wells plates.

### Flow cytometry

NSC34 cells and C2C12 cells were plated in a 12-well plate (respectively 90,000 cells/well and 65,000 cells/well) and transfected with pDS-RED-monomer and GFP-TDP25. After 48 hrs, cells were harvested and rinsed in PBS. 10,000 events per samples were analysed, and gating based on FSC-H and SSC-H parameters was performed to select living cells. Green fluorescence was recorded at 488 nm and red fluorescence was recorded at 594 nm.

### Fractionation

Both cell lines were plated in 6-well plate (NSC34 200,000 cells/well; C2C12 130,000 cells/well). 48 hrs after transfection cells were harvested and centrifuged at 100 g for 5 min at 4 °C. Cells were then diluted in 100 µL PBS (Euroclone) added with protease inhibitor cocktail (Sigma-Aldrich), sonicated (3 hits at lowest intensity) and centrifuged fat maximum speed for 15 min at 4 °C. Supernatant (corresponding to PBS soluble fraction) was then transferred in a new tube and pellet was rinsed in 100 µL of 1% TRITON X-100 (Sigma-Aldrich) solution in PBS. Suspension was slightly sonicated and centrifuged at maximum speed for 15 min at 4 °C. The supernatant (that corresponds to TRITON X-100 soluble fraction) was placed in a new tube. Pellet was rinsed in 100 µL of a solution of PBS and 5% SDS (Sigma-Aldrich) sonicated and centrifuged to obtain SDS soluble fraction. The SDS soluble fraction was transferred in a new tube and the pellet was rinsed in 100 µL of a solution of 88% formic acid (FA) (Sigma-Aldrich) in PBS, sonicated and centrifuged with same protocol used to obtain TRITON X-100 and SDS soluble fraction. The FA soluble fraction was transferred in a new tube and the remaining pellet was discarded^11,13^. PBS soluble fraction and TRITON X-100 soluble fraction were quantified using bicinchoninic acid (BCA) (Euroclone) assay. SDS soluble fraction and FA soluble fraction were loaded as equal volume to PBS soluble fraction.

### NP40 soluble insoluble extraction

Cells were plated in a 12-well plate (NSC34 90,000 cells/well; C2C12 65,000 cells/well). 48 hrs after transfection cells were lysed passing each sample with syringe in the Nonidet P40 (NP-40) buffer (150 mM NaCl (Sigma-Aldrich); 20 mM TrisBase (Sigma-Aldrich); Nonidet P-40 (NP-40) 0.5% (Sigma-Aldrich); 1.5 mM MgCl_2_ (Sigma-Aldrich); Glycerol 3% (Sigma-Aldrich), pH 7.4), with protease inhibitors cOmplete (Roche) and 1,4-Dithiotreitol 1 Mm (DTT; Sigma Aldrich) to eliminate disulphide bonds. Samples were then centrifuged for 15 min at maximum speed and the supernatants (the NP-40 soluble fraction) were transferred in new tubes while the pellets were rinsed in fresh NP-40 buffer, without protease inhibitor and DTT. NP-40 soluble fractions were quantified with bicinchoninic acid (BCA) assay (Euroclone). Each NP-40 insoluble fraction has been loaded as equal volume to NP-40 soluble fraction.

### Filter retardation assay

Filter retardation assay (FRA) is based on filtration of proteins on a cellulose acetate membrane with pores of 0.22 µm by a vacuum applied at a Bio-Dot SF Microfiltration Apparatus (Bio-Rad, Hercules, CA, USA). FRA allows the detection of insoluble protein species larger than the pores of the membrane. The following amounts of proteins were loaded: 1.5 µg of NSC34 PBS and NP-40 soluble extracts (NP-40 insoluble extracts were loaded as equal volume), 6 µg of C2C12 PBS and NP-40 soluble extracts (NP-40 insoluble extracts were loaded as equal volume). After vacuum filtration proteins were fixed on the membrane with 20% methanol and then the membrane was incubated for 1 hr at RT in blocking solution (5% of dried non-fat milk (Euroclone) in TBS-T 1X) and then with primary antibody for 1 hr at RT. After two washes with TBS-T 1X the membrane was incubated with the secondary antibody (find incubation timing and antibody dilution below). After three washes with TBS-T 1X at RT membrane was incubated with ClarityTM Western ECL Blotting Substrate (Bio-Rad) to reveal the signal. Images were acquired using a ChemiDoc XRS System (Bio-Rad)^36^.

### Western blot

Protein samples were loaded on 10%, 12% or 15% polyacrylamide gels, depending on the resolving power needed. 15 µg of PBS, TRITON X-100 and NP-40 soluble extracts were loaded for both cell lines (SDS and FA soluble extracts were loaded as equal volume to PBS soluble extracts; insoluble NP-40 extracts were loaded as equal volume to NP-40 soluble extracts).

Proteins were transferred to nitrocellulose membrane (Bio-Rad) with a TransBlot (Bio-Rad) following manufacturer’s instructions. Membranes were then incubated with blocking solution (5% of dried non-fat milk (Euroclone)) for 1 hr at RT and then with primary antibody at 4 °C overnight. After two washes, membranes were incubated with the HRP-conjugated secondary antibody diluted in TBS-T 1X for 1 hr at RT. Signal was detected with ChemiDoc XRS System (Bio-Rad) after incubation with ClarityTM Western ECL Blotting Substrate (Bio-Rad). Antibody description and dilution are described below.

### DSG cross-linking and nucleus/cytoplasm fractionation

Cells were plated in a 12-well plate (NSC34 90,000 cells/well; C2C12 65,000 cells/well). After transfection, cells were rinsed in PBS added with DSG 500 μM (Sigma-Aldrich; 80424). After incubation at RT for 30 min, reaction was stopped by adding Tris-base (Sigma-Aldrich) for 15 min at room temperature (RT) (at a final concentration of 20 mM). DSG crosslinking was followed by nucleus/cytoplasm fractionation that was performed as previously published^37^. Briefly, cells were centrifuged at 100 g for 10 min at 4 °C and cell pellet was rinsed in cell lysis buffer. After incubation in ice for 5 min, samples were centrifuged at 3,000 g for 15 min. The supernatant, cytosolic fraction, was separated by the pellet, nuclear fraction. Cytosolic fraction was quantified by BCA, while nuclear fraction was loaded as equal volume.

### Antibodies

The following primary antibodies were used: HRP-conjugate anti-GFP antibody (Vector Laboratories, Burlingame, CA, USA; MB-0712) for TDPs detection in FRA and WB (1:10,000 for NSC34; 1:2,000 for C2C12); mouse monoclonal anti-FLAG (Sigma-Aldrich; F1804) for TDPs detection in FRA (1:2000) and WB (1:1,000); mouse monoclonal anti-TDP-43 (Proteintech; 12892) (1:1,000); home-made rabbit polyclonal anti-HSPB8 (1:2,000); home-made polyclonal anti-Bag3 (1: 3,000); rabbit polyclonal anti-HA (Santa Cruz Biotechnology; sc-7392) (1:500); goat polyclonal anti-ACTIN (Santa Cruz Biotechnology; sc-1616) (1:1,000); rabbit polyclonal anti-GAPDH (Santa Cruz Biotechnology; sc-25778) (1:1,000); rabbit polyclonal anti-p62 (Sigma-Aldrich; P0067) (1:2,000); rabbit polyclonal anti-LC3 (Sigma-Aldrich; L8918) (1:2,000); mouse monoclonal anti-α-tubulin (Sigma-Aldrich; T6199) (1:2,000).

The following secondary antibodies were used: goat anti-rabbit HRP-conjugate secondary antibody (Santa Cruz Biotechnology; sc-2004); goat anti-mouse HRP-conjugate secondary antibody (Santa Cruz Biotechnology; sc-2005); donkey anti-goat HRP-conjugate secondary antibody (Santa Cruz Biotechnology; sc-2020).

### Immunofluorescence analysis

Cells were seeded on coverslips in a 24-well plate (NSC34 70,000 cells/well; C2C12 50,000 cells/well). 48 hrs after transfection, cells were fixed using paraformaldehyde 4% (30 min at 37 °C) and methanol (10 min at RT). Cells were permeabilized using a solution of TRITON X-100 10% diluted 1:5,000 in PBS. After the incubation of 1 hr in blocking solution, cells were incubated with primary antibody overnight at 4 °C. The following primary antibodies were used: mouse monoclonal anti-FLAG (Sigma-Aldrich; F1804) (1:500); home-made rabbit polyclonal anti-HspB8 (1:500 kindly provided by Dr. Landry Centre of Recherche Cancerologie, University of Laval, Canada), home-made rabbit polyclonal anti-Bag3 (1:500 kindly provided by Dr. Landry), rabbit polyclonal anti-HA (1:250, Santa Cruz, sc-805), rabbit polyclonal anti-p62 (Sigma-Aldrich; P0067) (1:500); rabbit polyclonal anti-LC3 (Sigma-Aldrich; L8918) (1:500). After two washes with PBS, cell were incubated for 1 hr at RT with goat anti-rabbit 549 Alexa (1:1,000, Life Technologies, Thermo Fischer, Cod. A-11012) or goat anti-mouse 549 Alexa (1:1,000, Life Technologies, Thermo Fischer, Cod. A-11020). Nuclei were stained with DAPI (1:10,000 in PBS).

Coverslips were then mounted on a support using MOWIOL solution. Images were acquired using LSM510 Meta system confocal microscope (Zeiss, Oberkochen, Germany) and processed with the Aim 4.2 software (Zeiss).

### Fluorescence quantification

Cells were seeded on coverslips in a 24-well plate (NSC34 70,000 cells/well; C2C12 50,000 cells/well). 48 hrs after transfection cells were fixed using paraformaldehyde 4% (30 min at 37 °C) and methanol (10 min at RT). After immunofluorescence procedure described above, ten fields per sample were acquired using Zeiss Axioskop2 plus microscope equipped TCH-5.0ICE digital camera at 40X and three biological samples were analysed. Every image was acquired with the same exposure parameters and was analysed using ImageJ. Each transfected cell per field was masked and fluorescence per cell was measured.

### Aggregates counts

Cells were seeded on coverslips in a 24-well plate (NSC34 70,000 cells/well; C2C12 50,000 cells/well). 48 hrs after transfection, or after treatments, cells were fixed using paraformaldehyde 4% (30 min at 37 °C) and methanol (10 min at RT). After immunofluorescence procedure described above, ten fields per sample were acquired using Zeiss Axioskop2 plus microscope equipped TCH-5.0ICE digital camera at 40X and three biological samples were analysed.

For each experiment, the same threshold was applied at every image and the number of dots per cell was counted. GFP-TDP-25 dots composed of more than 1,000,000 pixels were called big aggregates, while dots composed of less than 1,000,000 pixels were called small aggregates.

### Statistical analysis

Each condition was carried out in three biological replicates, and each experiment was performed three times. PRISM (ver. 6.0) software (Graph- Pad Software, La Jolla, CA, USA) was used to calculate mean and standard deviation (SD) for each replicate. All data are presented as mean ± SD and one-tailed unpaired Student’s t test was used to compare two groups.

### Data availability

The datasets generated and/or analysed during the current study are available from the corresponding author on reasonable request.

## Results

TDP-43 is a DNA and RNA binding protein, that behaves aberrantly in ALS cases. The biochemical behavior and clearance of this protein and its ALS-associated fragments are still largely uncharacterized in muscle cells, which are known to be involved in disease onset and progression. Here, we compared the behavior of these proteins in immortalized mouse motoneuronal NSC34 cells (i-motoneurons) and stabilized mouse myoblasts C2C12 cells (s-myoblasts), focusing on possible pathways capable to increase the degradation of misfolded and aggregation-prone proteins.

### Biochemical behavior of TDP-43 and sALS-associated fragments in motoneuronal and muscle cells

**Figure 1:**
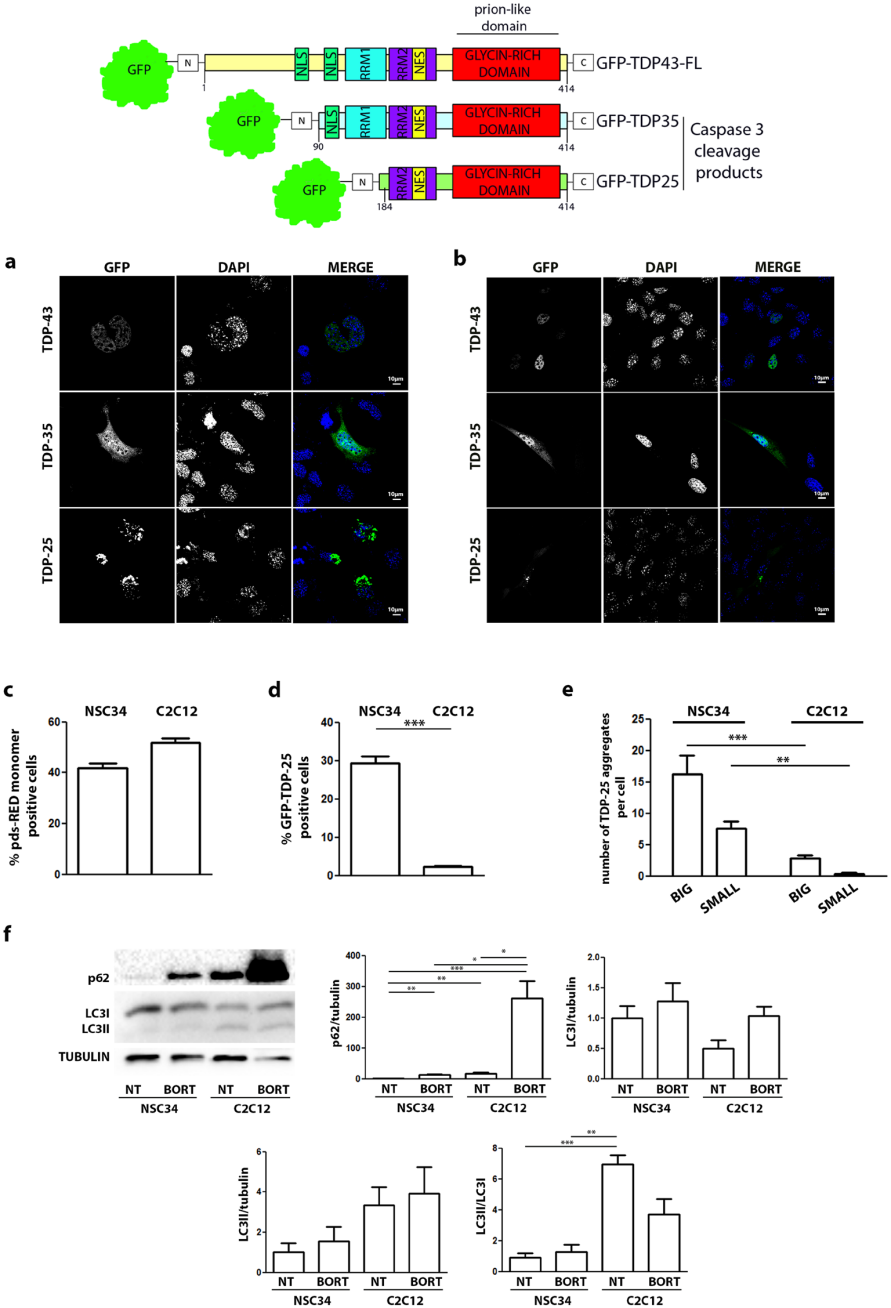
TDP-43 variants show different behavior in NSC34 and C2C12 cells. (Upper Panel) schematic representation of TDPs variants used in this study. (**a**,**b**) Confocal microscope analyses. NSC34 (**a**) or C2C12 (**b**) cells were transiently transfected with GFP-TDP-43, GFP-TDP-35, GFP-TDP-25 plasmids. Nuclei were stained with DAPI. Images were acquired using confocal microscope with 63X magnification. (**c**,**d**) Fluorescent flow cytometry analysis. NSC34 and C2C12 were transiently transfected with pDS-RED-monomer and GFP-TDP-25: (**c**) percentage of cells displaying red fluorescence and (**d**) percentage of red fluorescent cells displaying green fluorescence. (**e**) Aggregates count. NSC34 and C2C12 were transiently transfected with GFP-TDP-43, GFP-TDP-35, GFP-TDP-25. Big aggregates: dots composed of more than 1,000,000 pixels. Small aggregates: dots composed of less than 1,000,000 pixels. (**f**) Degradative systems activation in NSC34 and C2C12. NSC34 and C2C12 were treated with Bortezomib 200 nM. WB (15% polyacrilamide gel) shows p62, LC3I and LC3II levels. Graphics show quantification of p62 protein levels normalized on tubulin, quantification of LC3I protein levels normalized on tubulin, quantification of LC3II protein levels normalized on tubulin, LC3II/LC3I ratio (full-length blots/gels are presented in Supplementary Figure S4).

Initially, we tested the intracellular distribution of overexpressed full length TDP-43 and its sALS-associated fragments of 35 kDa (TDP-35) and 25 kDa (TDP-25) (see schematic representation of plasmids in Fig. 1). We also compared their biochemical behavior in i-motoneurons and s-myoblasts.

In i-motoneurons, we noted that TDP-43 entirely localized in the nucleus, while no visible aggregates were detectable in the cytoplasm. The TDP-35 fragment partially mislocalized in the cytoplasm, eventually forming aggregates in the cytoplasm. The TDP-25 fragment was completely excluded from the nucleus and fully confined in the cytoplasm, forming aggregates with an irregular shape, observed both in the perinuclear region and in the neurites (Fig. 1a). When we analysed the various overexpressed TDP forms in s-myoblasts (C2C12 cells) (Fig. 1b), we noted a similar biochemical behavior. In fact, in s-myoblasts, we found that, TDP-43 retains its nuclear localization as seen in i-motoneurons, and no visible cytoplasmic aggregates were detectable. TDP-35 partially mislocalized from nucleus to cytoplasm forming few cytoplasmic aggregates, while TDP-25 fully mislocalized in the cytoplasm forming perinuclear aggregates. The different behavior of the TDP-25 fragment is not related to changes of the transfection efficiency (CMV promoter both for ds-RED and TDPs constructs), which was comparable in the two cells types (Fig. 1c), but rather to differential overall turnover of the TDP-25 fragment (Fig. 1d). We also observed that TDP-25 aggregates present in s-myoblasts were much lower both in number and in size when compared to i-motoneurons (Fig. 1e). In general, the number of TDP-25 “aggregates per cells” found in s-myoblasts was much lower than that detected in i-motoneurons (Fig. 1e). These evidences could be explained by the higher degradative power of s-myoblasts (C2C12 cells) which seems to be higher than that of i-motoneurons (NSC34 cells), since we already shown that despite a lower expression rate of LC3 in C2C12 than in NSC34 cells, the activation of the LC3-I protein to its lipidated form LC3-II associated to autophagosomes is much higher in C2C12 than in NSC34 cells^32^. Indeed, in Fig. 1f it is possible to appreciate that the conversion LC3-I to LC3-II (LC3-II/LC3-I ratio) is much higher in C2C12 than in NSC34 cells. Moreover, the levels of SQSTM1/p62 are significantly higher in s-myoblasts than in i-motoneurons, indicative of a higher activity of autophagy in i-motoneurons.

**Figure 2:**
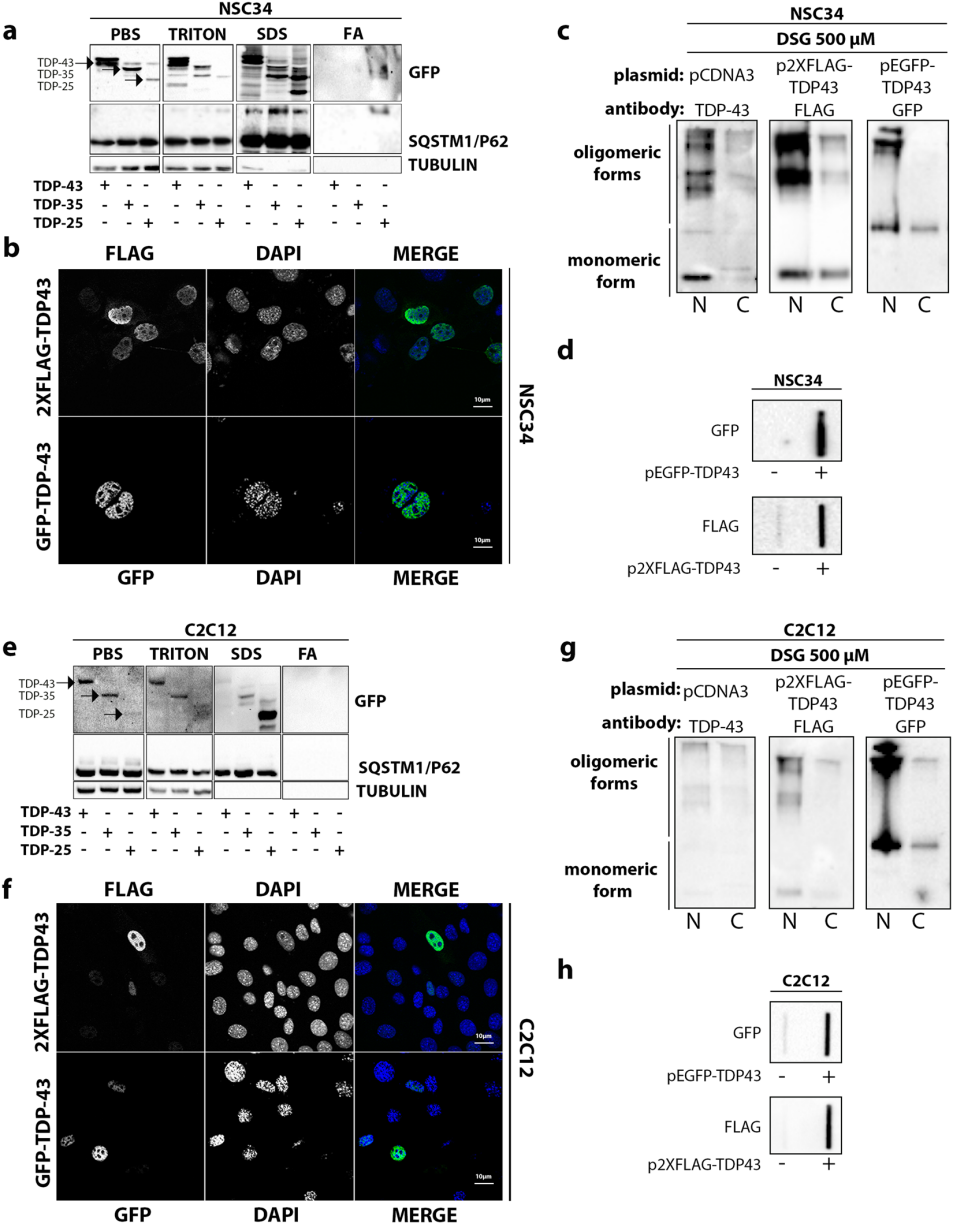
TDP-43 variants behavior in NSC34 and C2C12 cells. (**a**) Fractionation. NSC34 cells were transiently transfected with GFP-TDP-43, GFP-TDP-35, GFP-TDP-25. Western blot (WB) shows PBS, TRITON X-100, SDS and formic acid (FA) extracts, each set of sample loaded on a separate 10% polyacrylamide gel. Tubulin was used as loading control (full-length blots/gels are presented in Supplementary Figure S5). (**b**) Confocal microscope analysis. NSC34 cells were transiently transfected with GFP-TDP-43, or p2XFLAG-TDP-43. Nuclei were stained with DAPI. Images were acquired using confocal microscope with 63X magnification. (**c**) Nucleus/cytoplasm fractionation: NSC34 cells were transiently transfected with pcDNA3, GFP-TDP-43, or p2XFLAG-TDP-43. WB shows nuclear and cytoplasmic fractions, each set of sample loaded on a different 10% polyacrylamide gel (full-length blots/gels are presented in Supplementary Figure S5). (**d**) Filter retardation assay performed on PBS extracts of NSC34 transfected with pEGFP-TDP-43 and p2XFLAG-TDP-43 (full-length blots/gels are presented in Supplementary Figure S5). (**e**) Fractionation. C2C12 cells were transiently transfected with GFP-TDP-43, GFP-TDP-35, GFP-TDP-25. WB shows PBS, TRITON X-100, SDS and formic acid (FA) extracts, each set of sample loaded on a separate 10% polyacrylamide gel. Tubulin was used as loading control (full-length blots/gels are presented in Supplementary Figure S6). (**f**) Confocal microscope analysis. C2C12 cells were transiently transfected with GFP-TDP-43, or p2XFLAG-TDP-43. Nuclei were stained with DAPI. Images were acquired using confocal microscope with 63X magnification. (**g**) Nucleus/cytoplasm fractionation: C2C12 were transiently transfected with pcDNA3, GFP-TDP-43, or p2XFLAG-TDP-43. WB shows nuclear and cytoplasmic fractions, each set of sample loaded on a different 10% polyacrylamide gel (full-length blots/gels are presented in Supplementary Figure S6). (**h**) Filter retardation assay performed on PBS extracts of C2C12 transfected with pEGFP-TDP-43 and p2XFLAG-TDP-43 (full-length blots/gels are presented in Supplementary Figure S6).

Fractionation study performed using selected detergents with increasing solubility power (PBS, Triton, SDS, FA) showed that, both in i-motoneurons (Fig. 2a) and in s-myoblasts (Fig. 2e), the full-length (FL) form of TDP-43 did not accumulate as SDS-resistant species in FA fraction (Fig. 2a,e). These data are in line with the lack of mislocalization and aggregation evaluated in IF microscopy, which is similar for TDP-43 proteins with different tags (Fig. 2b,f) in both cell types. The presence of TRITON-resistant TDP-43 species in SDS fraction (Fig. 2a,e) also suggested that the FL TDP-43 could generate multimeric species similar to those recently reported by Afroz and Coll.^37^. These findings indicated that these species could represent a physiological state of the FL TDP-43 proteins, which is able to oligomerize via a highly positively charged N-terminal domain (NTD) forming dynamic solenoid-like structures capable to self-associate and interact with target nucleic acids^37^. In order to provide further evidences supporting the oligomerization capability of TDP-43 in our conditions, and to avoid the influence of various N-terminal tags on TDP-43, we performed nuclear-cytoplasmic extraction, preceded by the DSG-mediated cross-link of multimeric TDP-43 species, on the endogenous TDP-43, or after overexpression of the 2xFLAG-TDP-43 and GFP-TDP-43. Apart from the different immunoreactivity noted due to the use of different antibodies (anti-TDP-43, anti-FLAG, anti-GFP), overall the data showed that TDP-43 (independently from the tag utilized) can be purified in its physiological oligomeric state both in i-motoneurons (Fig. 2c) and s-myoblasts (Fig. 2g) as reported by Afroz^37^. To prove the existence of these oligomeric species, we adopted the Filter Retardation Assay (FRA) technique, and found that TDP-43 was retained in acetate cellulose membrane (Fig. 2d,h), independently from the tag utilized, since very similar data have been obtained using a TDP-43 tagged with GFP or the very small 2xFLAG (Fig. 2d,h).

Interestingly, TDP-35 and TDP-25, which lack the functional NTD, but retain the unstructured “prion-like” C-terminal domain of the TDP-43 precursor, are able to aberrantly localize and aggregate in the cytoplasm of both cell types (Fig. 1a,b) even if at different levels. In the solubility assay, we found that only the TDP-25 generated SDS-resistant species in FA fraction in i-motoneuron (Fig. 2a, upper inset), while in s-myoblasts this fragment was the mostly represented TDP species resistant to TRITON X-100 (Fig. 2b, upper inset).

Therefore, TDP-43 and its 35 kDa fragment are mainly in a soluble form in motoneurons, while TDP-25 can form SDS-resistant inclusions, but only in i-motoneurons it was particularly enriched in this fraction. In SDS fraction, we also detected TDP species characterized by a molecular weight (M.W.) lower than expected, suggesting that all TDPs can be further cleaved in both cell types.

In addition, we found that also SQSTM1/p62, an autophagic receptor that internalizes substrates in autophagosomes, was detectable in large amounts in all fractions isolated from TDP-43 transfected cells, with the exception of the FA-fraction (Fig. 2a,b, middle insets). Specifically, in i-motoneurons (Fig. 2a, middle inset) an upshift of the SQSTM1/p62 to high M.W. forms was observed in the SDS-fraction associated to all TDPs forms analysed. In FA-fraction, the SQSTM1/p62 was detectable only in its high M.W. form in i-motoneurons, paralleling the accumulation of TDP-25 (Fig. 2a,b, middle insets), suggesting that TDP-25 SDS-resistant species co-segregated specifically with SQSTM1/p62. By measuring protein levels, we found no significant variation of SQSTM1/p62 in all condition tested (Figure S1a), suggesting that the SDS-resistant form of SQSTM1/p62 is a small fraction specifically sequestered by TDP-25 in i-motoneurons. Indeed, by co-transfecting GFP-TDP-25 and mCherry-p62 we found a perfect co-localization of the two proteins in i-motoneurons (NSC34 cells), while in s-myoblasts (C2C12 cells) this co-localization was absent, but the two proteins were found to be adjacent both in an aggregated form (Figure S1a). However, neither in NSC34 nor in C2C12 cells, TDP-25 over-expression induced changes in SQSTM1/p62 levels (Figure S1b). This suggests the existence of different interaction between the TDP-25 and SQSTM1/p62 in the two cell types, which may have an impact on the clearance of TDP-25 species at basal levels in NSC34 and C2C12 cells.

### The TDPs clearance in motoneuronal and muscle cells

Since the TDP-25 fragment is the one found in ALS-patients inclusions^10^, which presents the highest propensity to aggregate among the TDPs species considered in this study, and, as mentioned above, it lacks the entire nuclear localization signal (NLS) retaining the nuclear export signal (NES) and only the second RNA recognition motif (RRM-2) (see cartoon in Fig. 1), we focused our attention mainly on its biochemical behavior in the two cell types considered. To study the clearance of TDP-25 insoluble species in motoneuronal and muscle cells, we selectively inhibited the two major degradative pathways: the ubiquitin proteasome system (UPS) and autophagy. Because of the complexity of these experiments, to better analyze TDP-25 solubility, we decided to not utilize the four different detergents, but selected one with intermediate detergent capabilities: the non-ionic non-denaturing detergent NP-40. This detergent has power comprised between TRITON and SDS. We found that both in i-motoneurons (Figure S2a) and s-myoblasts (Fig. S2b) the TDP-25 species retained in the SDS-soluble fractions were equally retained in the NP-40 insoluble fractions.

**Figure 3:**
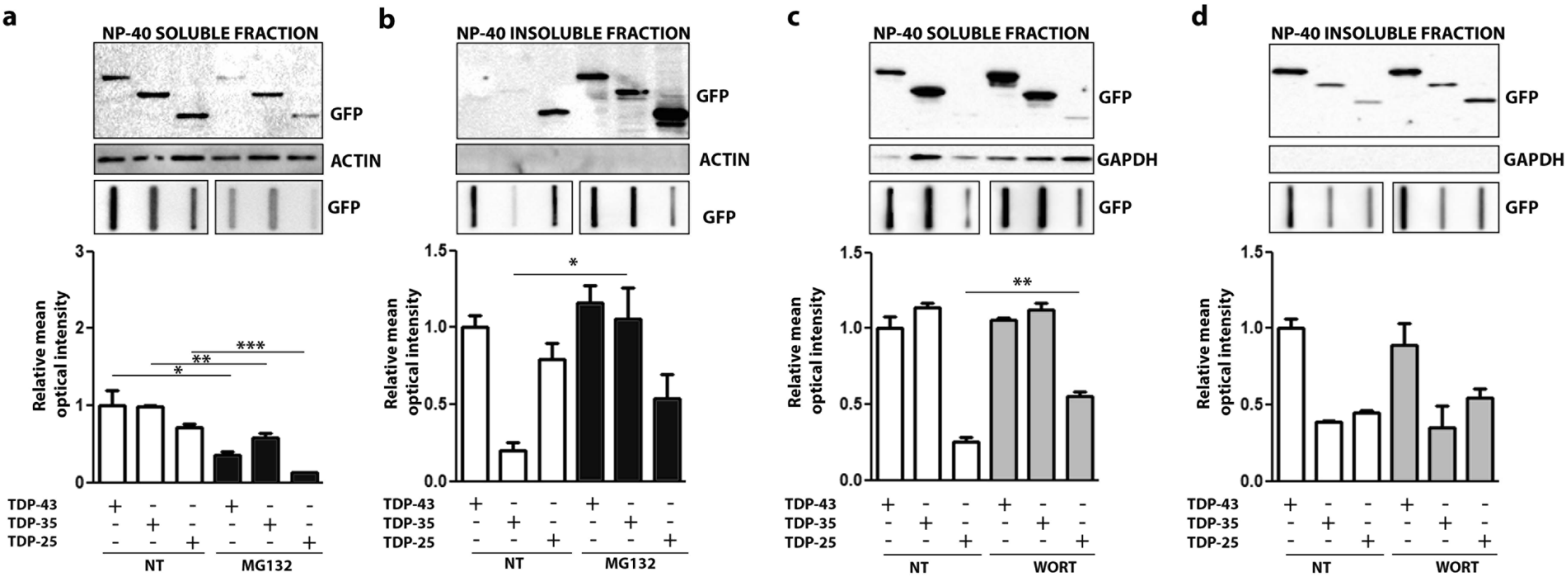
Degradative systems involvement in NSC34 cells with GFP-TDPs species. NSC34 transiently transfected with GFP-TDP-43, GFP-TDP-35 and GFP-TDP-25 were treated for 10 hrs with MG132 (10 µM) or for 36 hrs with Wortmannin (50 nM). (**a**,**c**) Panel shows NP-40 soluble extracts WB analysis (upper inset, 12% polyacrylamide gels) (full-length blots/gels are presented in Supplementary Figure S7), NP-40 soluble extracts FRA analysis (middle inset) (full-length blots/gels are presented in Supplementary Figure S7) and quantification of NP-40 soluble extracts FRA assay (*p < 0.05; **p < 0,01; ***p < 0.001) (lower inset); (**b**,**d**) Panel shows NP-40 insoluble extracts WB analysis (upper inset, 12% polyacrylamide gels) (full-length blots/gels are presented in Supplementary Figure S7), NP-40 insoluble extracts FRA analysis (middle inset) (full-length blots/gels are presented in Supplementary Figure S7) and quantification of NP-40 insoluble extracts FRA assay (*p < 0.05) (lower inset).

We evaluated the role of degradative systems in the clearance of the TDP-25, using the FL TDP-43 and the TDP-35 fragment as control. Figure 3a shows that in i-motoneurons the proteasome inhibition with MG132 leads to a modification of the NP-40 solubility of all TDP forms studied. In fact, both in WB (upper inset) and in FRA (lower inset) the levels of NP-40 solubilized species decreased after proteasome inhibition (Fig. 3a); only the TDP-35 form was found significantly increased in the fraction resistant to NP-40 (Fig. 3b, see also graph bars quantification of FRA), while the TDP-43 and TDP-25 NP-40 insoluble fraction were found increased and detectable in WB after proteasome inhibition. Notably, as expected^11^ autophagy inhibition with Wortmannin resulted in a mild increase of the overall TDP-25 levels (Fig. 3c) in NP-40 soluble fraction analysed in FRA, while it remained unchanged in NP-40 insoluble fraction analysed in WB and in FRA.

**Figure 4:**
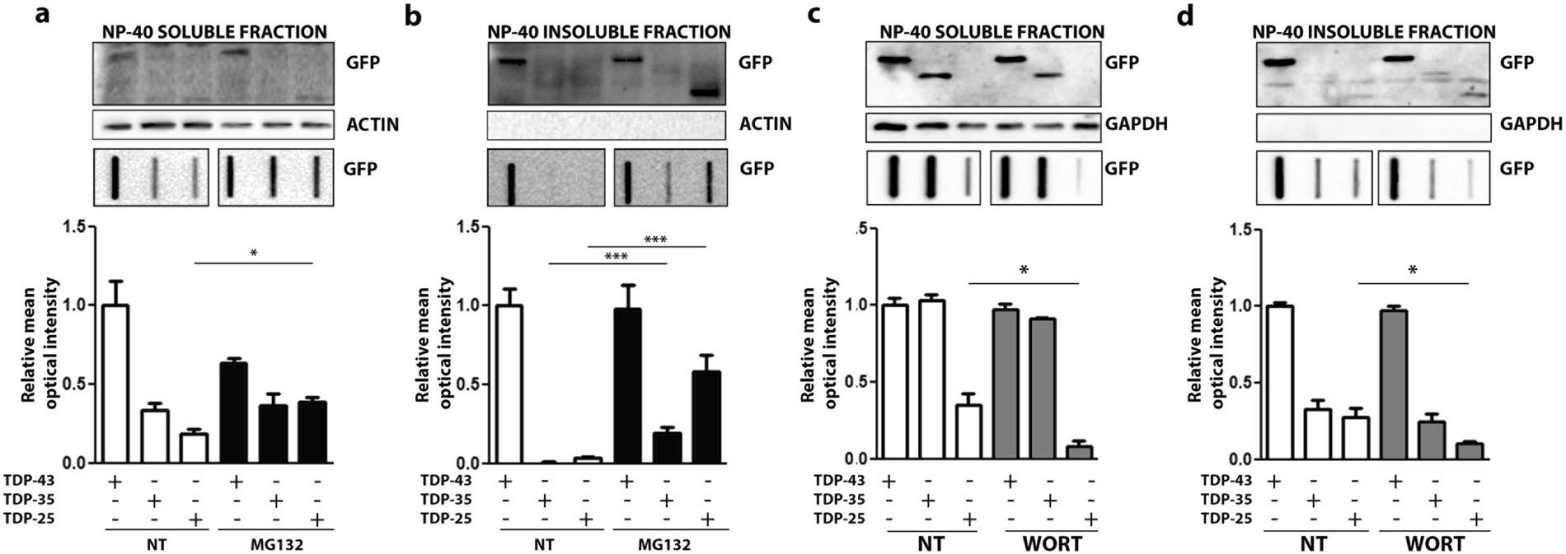
Degradative systems involvement in C2C12 with GFP-TDPs species. C2C12 were transiently transfected with GFP-TDP-43, GFP-TDP-35 and GFP-TDP-25 and were treated for 10 hrs with MG132 (10 µM) or for 36 hrs with Wortmannin (50 nM). (**a**,**c**) Panels show NP-40 soluble extracts WB analysis (upper inset, 12% polyacrylamide gels) (full-length blots/gels are presented in Supplementary Figure S8), NP-40 soluble extracts FRA analysis (middle inset) (full-length blots/gels are presented in Supplementary Figure S7) and quantification of NP-40 soluble extracts FRA assay (*p < 0.05; **p < 0,01) (lower inset); (**b**,**d**) Panels show NP-40 insoluble extracts WB analysis (upper inset, 12% polyacrylamide gels) (full-length blots/gels are presented in Supplementary Figure S7), NP-40 insoluble extracts FRA analysis (middle inset) (full-length blots/gels are presented in Supplementary Figure S7) and quantification of NP-40 insoluble extracts FRA assay (*p < 0.05; **p < 0,01) (lower inset).

By analysing the biochemical behavior of the TDP-25 forms in s-myoblasts (C2C12 cells) (Fig. 4) before and after modulation of the degradative systems, we found that proteasome inhibition with MG132 leads to an overall increase in TDP-25 levels analysed in FRA in the NP-40 soluble fraction. In addition, both TDP-43 fragments were found increased in the NP-40 insoluble fraction analysed in FRA (Fig. 4b, see data on FRA in the lower panel and relative graph bars quantification). This effect was particularly dramatic in the case of TDP-25 fragment. Among the three TDPs forms studied, the FL TDP-43 was not sensitive to proteasome inhibition. As mentioned above, the FL TDP-43 physiologically forms stable homodimers that allow its interaction with target mRNA^37,38^. It is possible that the TDP-43 forms found in FRA, also in these extraction experiments, represent the physiological fraction of the protein as we proposed from the experiments reported in Fig. 2g. However, the data of this analysis strongly support that these physiological oligomeric forms are not processed by the proteasome. As in i-motoneurons autophagy has a minor effect, and specifically on TDP-25 fragment, which was found to be marginally decreased possibly because a previously described compensatory mechanism involving the proteasome^13^.

Overall these data indicate that, in both cell type, i-motoneurons and s-myoblasts, proteasome system is the main pathway actively involved in the degradation of the TDPs fragments.

### Modulation of the TDP-25 routing to the degradative system ameliorates its clearance both in motoneuronal and muscle cells

So far, we have shown that TDP-25 is the form mainly responsible for TDP-43 aggregation in motoneuronal and muscle cells and that its aggregation is much more pronounced in motoneuronal than in muscle cells. These data are in line with our previous observation performed using a mutant SOD1^32^. It might be possible that in muscle cells this is related to a more efficient routing system between the two major degradative systems.

The routing system controlling the fine equilibrium between proteasome and autophagy is based on the activity of nucleotide exchange factors (NEFs), acting as co-chaperones of HSC70/CHIP. BAG1 is the NEF which routes misfolded protein to proteasome, BAG3, in conjunction with HSPB8, routes misfolded proteins to autophagy^39–43^. Therefore, we tested whether the higher proteasome power detected in muscle cells compared to motoneuronal cells could be due to variation of the routing system.

**Figure 5:**
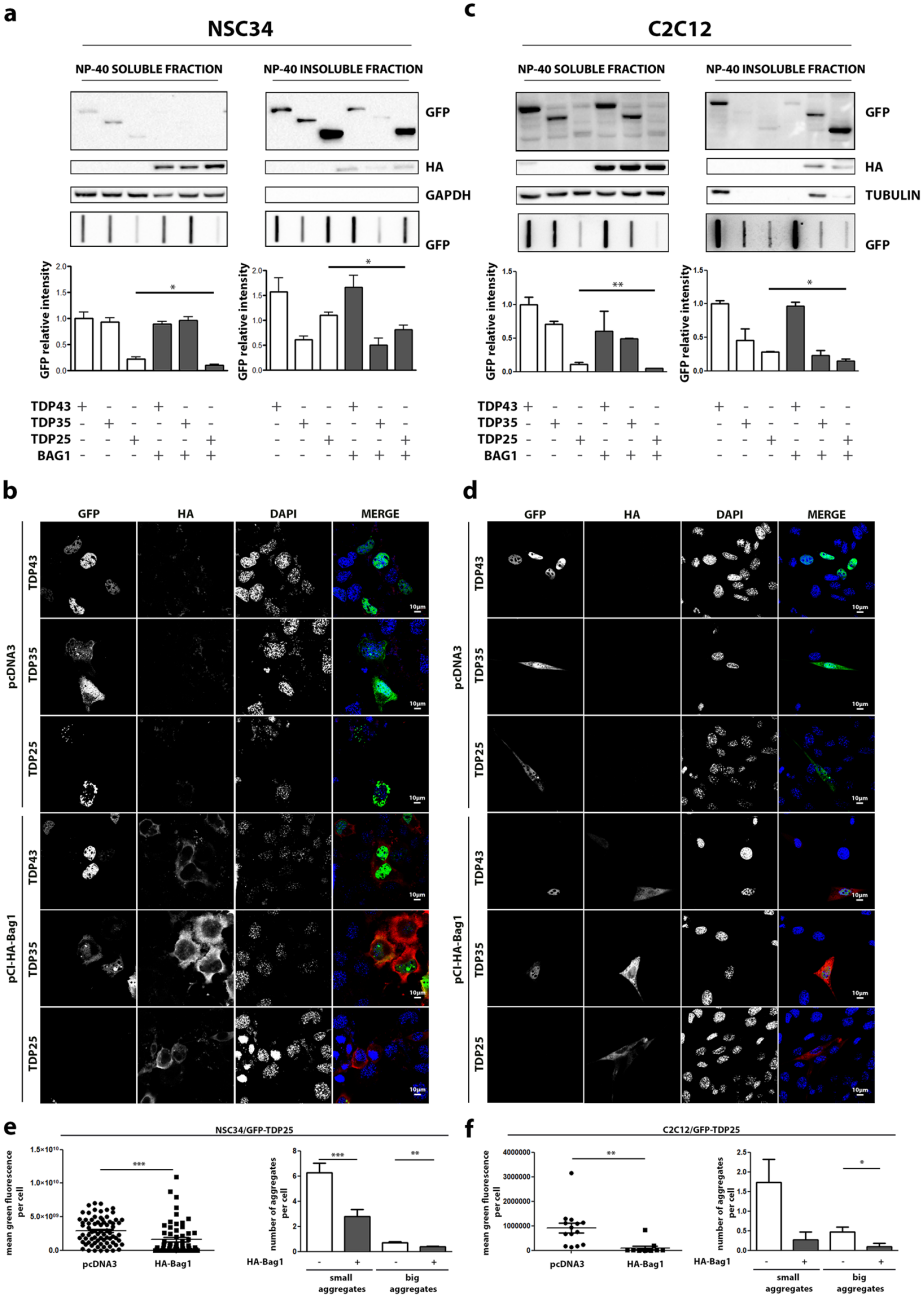
Proteasome re-routing is beneficial to GFP-TDP-25 aggregation. (**a**) NSC34 overexpressing GFP-TDPs, and co-transfected with pCI-HA-Bag1 or pcDNA3. Left panel: NP-40 soluble extracts WB analysis (upper inset, 12% polyacrylamide gels), NP-40 soluble extracts FRA analysis (middle inset) and quantification of NP-40 soluble extracts FRA analysis (*p < 0.05 *vs* GFP-TDP-25 co-transfected with pcDNA3) (lower inset). Right panel: NP-40 insoluble extracts WB analysis (upper inset, 12% polyacrylamide gels), NP-40 insoluble extracts FRA analysis (middle inset) and quantification of NP-40 insoluble extracts FRA analysis (*p < 0.05 *vs* GFP-TDP-25 co-transfected with pcDNA3) (lower inset). (**b**) NSC34 overexpressing GFP-TDPs, and co-transfected with pCI-HA-Bag1 or pcDNA3 analysed with confocal microscope. 63X magnification. Green: GFP-TDPs; Red: hBag1; nuclei: DAPI. (**c**) C2C12 overexpressing GFP-TDPs, and co-transfected with pCI-HA-Bag1 or pcDNA3. Left panel: NP-40 soluble extracts WB analysis (upper inset, 12% polyacrylamide gels), NP-40 soluble extracts FRA analysis (middle inset) and quantification of NP-40 soluble extracts FRA analysis (**p < 0.05 *vs* GFP-TDP-35 co-transfected with pcDNA3) (lower inset). Right panel: NP-40 insoluble extracts WB analysis (upper inset, 12% polyacrylamide gels), NP-40 insoluble extracts FRA analysis (middle inset) and quantification of NP-40 insoluble extracts FRA analysis (*p < 0.05 *vs* GFP-TDP-25 co-transfected with pcDNA3). (**d**) C2C12 overexpressing GFP-TDPs, and co-transfected with pCI-HA-Bag1 or pcDNA3 as control vector analysed with confocal microscope. 63X magnification. Green: GFP-TDPs; Red: hBag1; nuclei: DAPI. (**e**) Microscope analysis of NSC34 transiently co-transfected with GFP-TDP-25 and HA-Bag1 or pcDNA3. Right panel: quantification of green fluorescence per cell (***p < 0.001 *vs* pcDNA3). Left panel: quantification of aggregates per cell. Small aggregates: dots composed of less than 1,000,000 pixels (***p < 0.001 *vs* pcDNA3). Big aggregates: dots composed of more than 1,000,000 pixels (**p < 0.01 *vs* pcDNA3). (**f**) Microscope analysis of C2C12 transiently co-transfected with GFP-TDP-25 and HA-Bag1 or pcDNA3. Right panel: quantification of green fluorescence per cell (**p < 0.01 *vs* pcDNA3). Left panel: quantification of aggregates per cell. Small aggregates: dots composed of less than 1,000,000 pixels. Big aggregates: dots composed of more than 1,000,000 pixels (*p < 0.05 *vs* pcDNA3). Full-length blots/gels are presented in Supplementary Figure S8.

Firstly, we tested the effect of BAG1 overexpression on TDPs species in both cell models. The analysis performed confirmed the high accumulation of NP-40 insoluble TDP-25 in i-motoneurons (Fig. 5a), which were also confirmed by the IF (Fig. 5b) in which aggregates were detectable only with the exogenous expression of the TDP-25 fragment. In addition, the IF study showed that BAG1 overexpression completely blocked the formation of physically defined TDP-25 aggregates in i-motoneurons (Fig. 5b,e). By analyzing the effect of BAG1 on the biochemical properties of the various TDP forms, we found that, both in i-motoneurons and s-myoblasts, BAG1 overexpression had no effect on TDP-43 and TDP-35 NP-40 soluble or insoluble species measured by FRA. In the case of TDP-25, BAG1 overexpression caused a significant reduction of both NP-40 soluble and insoluble species detectable in FRA (Fig. 5a,c). Very interestingly, specifically in s-myoblast, we noted a robust increase of NP-40 insoluble, SDS-soluble TDP-25 species in WB (Fig. 5c, upper inset), which was not paralleled by an accumulation of the same fragment in FRA (Fig. 5c lower inset and graph bar quantification). As previously mentioned, IF analysis confirmed the presence of small TDP-25 aggregates in muscle cells, fully reverted by BAG1 overexpression as seen in i-motorneurons (Fig. 5d,f).

**Figure 6:**
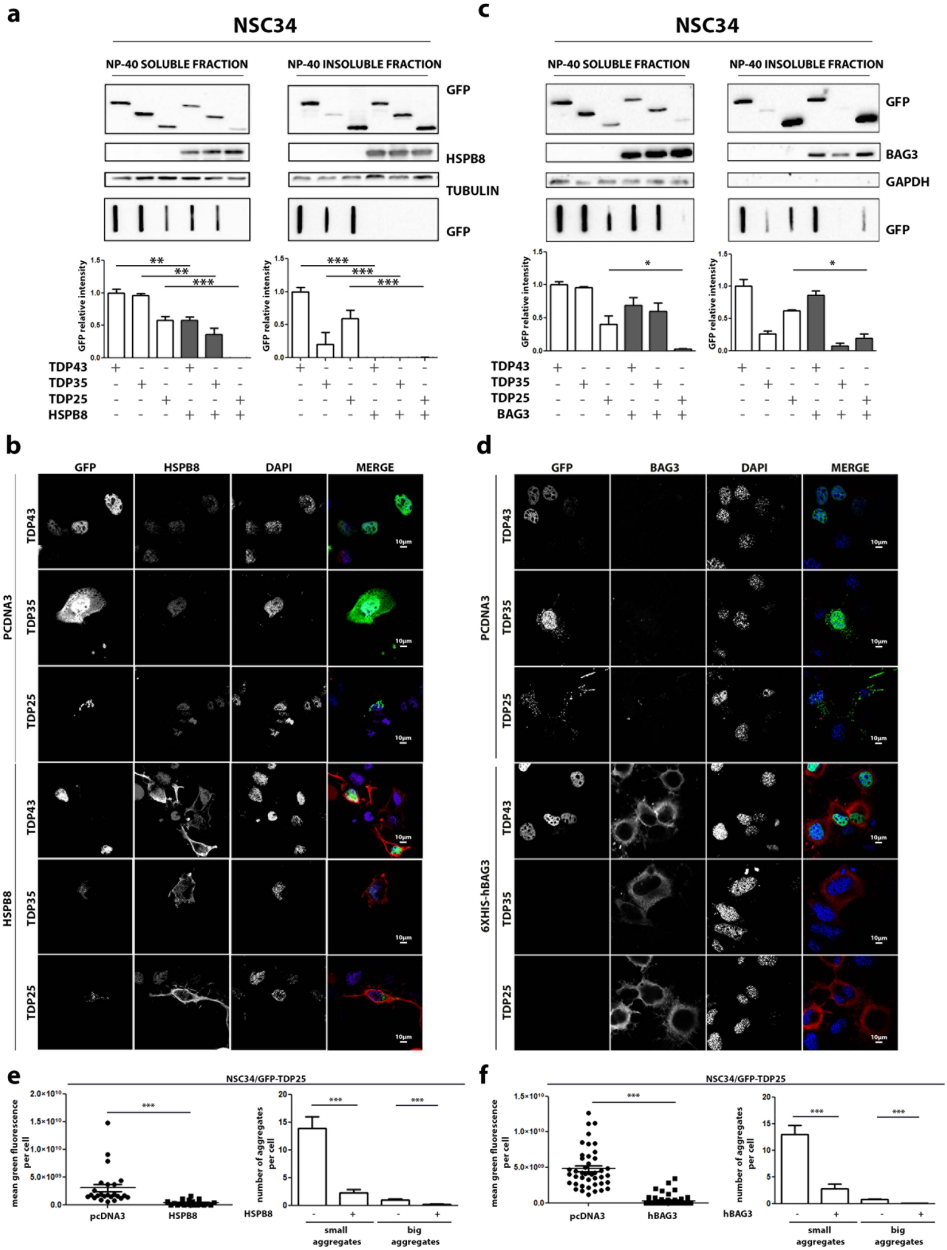
HSPB8 and its co-chaperone BAG3 counteract GFP-TDP-25 aggregation in NSC34. (**a**) NSC34 overexpressing GFP-TDPs, and co-transfected with pCI-hHSPB8 or pcDNA3. Left panel: NP-40 soluble extracts WB analysis (upper inset, 12% polyacrylamide gels) NP-40 soluble extracts FRA analysis (middle inset) and quantification of NP-40 soluble extracts FRA analysis (**p < 0.01; ***p < 0.001 *vs* relative GFP-TDPs co-transfected with pcDNA3) (lower inset). Right panel: NP-40 insoluble extracts WB analysis (upper inset, 12% polyacrylamide gels), NP-40 insoluble extracts FRA analysis (middle inset) and quantification of NP-40 insoluble extracts FRA analysis (***p < 0.001 *vs* GFP-TDP-25 co-transfected with pcDNA3) (lower inset). (**b**) NSC34 overexpressing GFP-TDPs, and co-transfected with pCI-hHSPB8 or pcDNA3 analysed with confocal microscope. 63X magnification. Green: GFP-TDPs; Red: hHSPB8; nuclei: DAPI. (**c**) NSC34 overexpressing GFP-TDPs, and co-transfected with pCI-6xHis-Bag3 or pcDNA3. Left panel: NP-40 soluble extracts WB analysis (upper inset, 12% polyacrylamide gels), NP-40 soluble extracts FRA analysis (middle inset) and quantification of NP-40 soluble extracts FRA analysis (*p < 0.05 *vs* GFP-TDP-25 co-transfected with pcDNA3) (lower inset). Right panel: NP-40 insoluble extracts WB analysis (upper inset, 12% polyacrylamide gels), NP-40 insoluble extracts FRA analysis (middle inset) and quantification of NP-40 insoluble extracts FRA analysis (*p < 0.05 *vs* GFP-TDP-25 co-transfected with pcDNA3) (lower inset). (**d**) NSC34 overexpressing GFP-TDPs, and co-transfected with pCI-6xHis-Bag3 or pcDNA3 analysed with confocal microscope. 63X magnification. Green: GFP-TDPs; Red: hBAG3; nuclei: DAPI. (**e**) Microscope analysis of NSC34 co-transfected with GFP-TDP-25 and pCI-hHSPB8 or pcDNA3. Right panel: quantification of green fluorescence per cell (***p < 0.001 *vs* pcDNA3). Left panel: quantification of aggregates per cell. Small aggregates: dots composed of less than 1,000,000 pixels (***p < 0.001 *vs* pcDNA3). Big aggregates: dots composed of more than 1,000,000 pixels (***p < 0.001 *vs* pcDNA3). (**f**) Microscope analysis of NSC34 co-transfected with GFP-TDP-25 and pCI-6xHis-Bag3 or pcDNA3. Right panel: quantification of green fluorescence per cell (***p < 0.001 *vs* pcDNA3). Left panel: quantification of aggregates per cell. Small aggregates: dots composed of less than 1,000,000 pixels (***p < 0.001 *vs* pcDNA3). Big aggregates: dots composed of more than 1,000,000 pixels (***p < 0.001 *vs* pcDNA3). Full-length blots/gels are presented in Supplementary Figure S9.

These data suggest that the potentiation of the proteasome arm of the routing system ameliorates TDP-25 degradation also in i-motoneurons, in which TDP-25 tends to accumulate in basal condition. We next examined whether HSPB8/BAG3 overexpression, which should enhance the routing to the autophagy arm of the degradative systems could also improve TDP-25 clearance. To this purpose, we initially evaluated in i-motoneurons whether the selective overexpression of HSPB8 and BAG3 modulate the clearance on the TDP forms in the two types of cells considered in this study. We have already demonstrated that HSPB8 overexpression promotes the degradation of TDPs insoluble species in motoneuronal cells^11^. The data confirmed that HSPB8 overexpression decreased both the NP-40 soluble and insoluble forms of all three TDP species detectable in FRA (Fig. 6a). These data were in line with the prodegradative HSPB8 effects on TDP forms evaluated in IF (Fig. 6b,e). Also, the overexpression of BAG3 generally recapitulated the observation obtained with its natural partner HSPB8 on NP-40 insoluble species, in which TDP-25 is specifically significantly reduced (Fig. 6c); also NP-40 insoluble species of TDP-35 showed a trend of reduction in presence of BAG3, but differences are not significative. However, the effect of BAG3 diverged on that of HSPB8 regarding NP-40 soluble forms observed by FRA, since in this case only TDP-25 was reduced, while the other two TDP forms remained unchanged (Fig. 6c,d,f).

The mechanism at the basis of the differences between these obligated partners remains obscure (Fig. 6c). Also in this case, the IF confirmed that BAG3 reduced the formation of physically defined TDP-25 aggregates in i-motoneurons; IF also showed a strong reduction in the presence of TDP-35 cells positive for BAG3.

**Figure 7:**
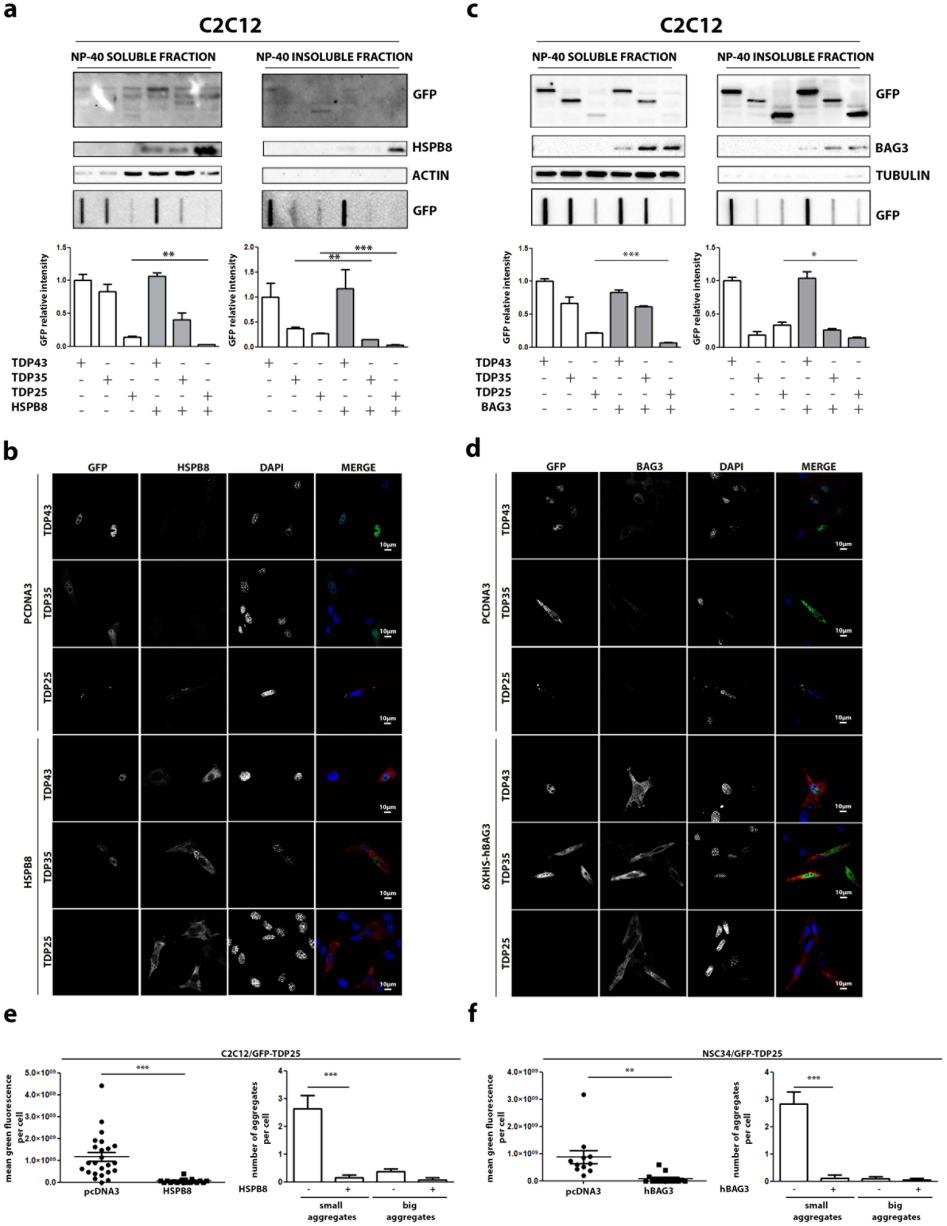
HSPB8 and BAG3 overexpression counteracted GFP-TDP-25 accumulation in C2C12. (**a**) C2C12 transiently overexpressing GFP-TDPs, and co-transfected with pCI-hHSPB8 or pcDNA3. Left panel: NP-40 soluble extracts WB analysis (upper inset, 12% polyacrylamide gels), NP-40 soluble extracts FRA analysis (middle inset) and quantification of NP-40 soluble extracts FRA analysis (**p < 0.01 *vs* GFP-TDP-25 co-transfected with pcDNA3) (lower inset). Right panel: NP-40 insoluble extracts WB analysis (upper inset, 12% polyacrylamide gels), NP-40 insoluble extracts FRA analysis (middle inset) and quantification of NP-40 insoluble extracts FRA analysis (**p < 0.01; ***p < 0,001 *vs* relative GFP-TDPs co-transfected with pcDNA3). (**b**) C2C12 transiently overexpressing GFP-TDPs, and co-transfected with pCI-hHSPB8 or pcDNA3 as control vector analysed with confocal microscope. 63X magnification. Green: GFP-TDPs; Red: hHSPB8; nuclei staining: DAPI. (**c**) C2C12 transiently overexpressing GFP-TDPs, and co-transfected with pCI-6xHis-Bag3 or pcDNA3. Left panel: NP-40 soluble extracts WB analysis (upper inset, 12% polyacrylamide gels), NP-40 soluble extracts FRA analysis (middle inset) and quantification of NP-40 soluble extracts FRA analysis (***p < 0.001 *vs* GFP-TDP-25 co-transfected with pcDNA3) (lower inset). Right panel: NP-40 insoluble extracts WB analysis (upper inset, 12% polyacrylamide gels), NP-40 insoluble extracts FRA analysis (middle inset) and quantification of NP-40 insoluble extracts FRA analysis (*p < 0.05 *vs* GFP-TDP-25 co-transfected with pcDNA3). (**d**) C2C12 transiently overexpressing GFP-TDPs, and co-transfected with pCI-6xHis-Bag3 or pcDNA3 analysed with confocal microscope. 63X magnification. Green: GFP-TDPs; Red: hBAG3; nuclei: DAPI. (**e**) Microscope analysis of C2C12 co-transfected with GFP-TDP-25 and pCI-hHSPB8 or pcDNA3. Right panel: quantification of green fluorescence per cell (***p < 0.001 *vs* pcDNA3). Left panel: quantification of aggregates per cell. Small aggregates: dots composed of less than 1,000,000 pixels (***p < 0.001 *vs* pcDNA3). Big aggregates: dots composed of more than 1,000,000 pixels. (**f**) Microscope analysis of C2C12 co-transfected with GFP-TDP-25 and pCI-6xHis-Bag3 or pcDNA3. Right panel: quantification of green fluorescence per cell (**p < 0.001 *vs* pcDNA3). Left panel: quantification of aggregates per cell. Small aggregates: dots composed of less than 1,000,000 pixels (***p < 0.001 *vs* pcDNA3). Big aggregates: dots composed of more than 1,000,000 pixels. Full-length blots/gels are presented in Supplementary Figure S11.

Then, we studied the role of HSPB8 and BAG3 on the clearance of the TDP forms in s-myoblast. In all tests performed, HSPB8 (Fig. 7a,b,e) and BAG3 (Fig. 7c,d,f) showed a pro-degradative activity very similar to that found in motoneuronal cells, both on NP-40 soluble and insoluble fraction, with the only exception of an increased response of the TDP-35 fragment to the action of HSPB8 in NSC34 (see Fig. 7a lower inset and graph bar quantification, to be compared with the same experimental set in Fig. 6a).

We next analysed whether the differentiation state of i-motoneurons (NSC34) and s-myoblast (C2C12) influences the clearance of the TDP species. Indeed, the degradative system contribution could change upon cell differentiation. NSC34 cells were differentiated by using retinoic acid (at 10 µM for 72 hrs) and C2C12 by using horse serum (see methods) (Figure S3a). Very interestingly, the pro-degradative effect of HSPB8 observed in undifferentiated cells was fully recapitulated also in differentiated cells (Figure S3b–e). Indeed, when HSPB8 was overexpressed in differentiated i-motoneurons, insoluble species of TDP-25 were significantly reduced (Figure S3b). Also in differentiated C2C12 HSPB8 produced the same trend of reduction of TDP-25 species, observed in undifferentiated C2C12. Finally, IF analysis revealed a reduction in the formation of TDP-25 insoluble species in both differentiated i-motoneurons and s-myoblasts (Figure S3d,e).

## Discussion

In recent years, several studies have suggested that alteration in skeletal muscles may contribute to the onset and progression of several types of ALS. Muscle has been directly involved as one of the primary targets of disease or indirectly, being affected by denervation and atrophy in ALS, thus less capable to produce factors exerting trophic protection on remaining motoneuronal cells in the spinal cord. Most of the experimental data suggesting the existence of a contribution of muscle cells to ALS have been obtained using SOD1-based animal and cellular models^16,17,19,22–24,32,44,45^. Interestingly, the overexpression of TDP-43 in transgenic mice results in alteration of skeletal muscle tissue, with increased accumulation of a RAB-GTPase activating protein (Tbc1d1), which aberrantly regulates the translocation of the Glucose 4 transporter (Glut4), leading to alteration of the insulin mediated glucose uptake^27^. Other studies have demonstrated that, in ALS patients, the muscle has a lower glycolytic activity and the energy requirement is supported by the metabolism of fatty acids^28^. TDP-43 pathology is indeed a multisystem proteinopathy, which may also appear as sporadic inclusion body myositis (IBM)^9^. Indeed, in cultured muscle cells and mouse skeletal muscle, TDP-43 acetylation appears to be a trigger to initiate the disease, promoting the phosphorylation and ubiquitination of TDP-43, which induces alteration at mitochondrial levels and activates an inflammatory response similar to that found in sporadic IBM^9^. Very recently, Cykowsky *et al*.^29^ found TDP-43 inclusions in selected muscle tissues derived from ALS patients, in particular in the axial skeletal muscles, paraspinus and diaphragm, while others, such as the quadriceps or the deltoid, were devoid of inclusions. This suggests that the clearance of the TDPs toxic species may significantly change in the different muscle types on the body in affected patients. These findings pave the way for further analysis on the biochemical behavior of TDP-43, and of its C-terminal ALS-associated fragments in muscle derived cells that may differ from motoneuronal cells.

In this study, we found that the TDP-25 fragment is the specie with the highest aggregation propensity, especially in motoneurons (although it also aggregates in muscle cells). The analysis of the intracellular distribution allowed us to determine that TDP-43 is in the nuclei of both motoneuronal and muscle cells, but TDP-35 partially mislocalizes in the cytoplasm forming few small aggregates, and TDP-25 is excluded from the nucleus and aggregates in the cytoplasm. The TDP-25 aggregates are characterized by an irregular shape and are much bigger than those generated by TDP-35. However, the number and size of these aggregates are reduced in muscle cells compared to motoneuronal cells.

Indeed, TDP-25 is found in formic acid resistant forms together with SQSTM1/p62 bodies (autophagic markers, that suggest a possible different contribution of autophagy in correctly degrading it), while TDP-43 and TDP-35 are more soluble than TDP-25. The overall biochemical behavior of these TDPs species in muscle cells overlaps that found in motoneuronal cells, except for the TDP-25, which is not present as SDS-resistant or FA-resistant form in muscle cells.

Thus, at least in the case of the most prone-to-aggregate species of TDP-43 (TDP-25), motoneurons may have a reduced degradative power compared to muscle cells. Interestingly, in motoneurons, all TDP forms are processed by the proteasome, while autophagy seems to be impaired specifically by TDP-25. In muscle cells, proteasome mediates the clearance of all TDPs forms, and when autophagy is blocked, TDP-25 is more rapidly degraded by the proteasome in muscle than in motoneuronal cells.

Collectively these data point to a more efficient routing system between proteasome and autophagy in muscle cells when compared to motoneuronal cells. These data parallel our previous observations showing the existence of a much efficient clearance of the mutant misfolded SOD1 in muscle cells, when compared to motoneuronal cells^32^, and the fact that mutant SOD1 aggregates alter proteasome function only in motoneuronal cells. Also in the case of SOD1, we found that muscle cells mainly base their clearance of misfolded species on the proteasome system, but accompanied by a very efficient autophagic system. Thus, it is possible that also for the mutant SOD1 the lack of aggregation in muscle is due to a better optimization of the routing system if compared to motoneuronal cells.

It is evident that much depends also on the state of aggregation of a given misfolded protein. Indeed, the aggregation process is a highly dynamic process, that starts with oligomerization of monomeric misfolded species and that ends with the formation of fibrillary structures. In our models, these insoluble structures are formed also in basal condition, even without degradative system failure. In this context, slowing down aggregation could be achieved by enhancing the activity of the chaperone network that can correctly and massively direct proteins to degradation. In association with HSP70 and CHIP1, BAG1 is mainly involved in misfolded proteins routing to proteasome. We demonstrated that BAG1 overexpression prevents the accumulation of the TDP-25 insoluble species in muscle cells. Thus, an increased proteasome activity enhances the TDP-25 clearance also in motoneurons. HSP70 can also drive proteins to autophagy when is associated with HSPB8/BAG3. Overexpression of either HSPB8 or BAG3 reduces the accumulation of all three TDPs proteins analysed, but in a slightly different manner, since BAG3 was less effective on TDP-25 and less effective on the soluble species of TDP-43 and TDP-35. Thus, selective BAG3-independent activities of HSPB8 may exist.

In conclusion, based on our data it appears that both motoneuronal and muscle cells are unable to properly degrade aggregation prone proteins, even if to different extent since muscles seem to have a more efficient degradative power. We demonstrated that in both type of cells, an optimization of the routing system to either proteasome or autophagy is sufficient to enhance the clearance of all TDP-43 fragments, slowing down their aggregation, oligomerization and also the presence of soluble monomeric species. In this context, boosting one or both systems may be an interesting therapeutic approach to counteract TDP-43 toxicity at different districts affected in ALS patients.

## Electronic supplementary material

Supplementary Figures and data
