# Supplementary material for: Tdp-25 Routing to Autophagy and Proteasome Ameliorates its Aggregation in Amyotrophic Lateral Sclerosis Target Cells
Source: Sci Rep. 2018 Aug 17;8:12390. doi: 10.1038/s41598-018-29658-2 (PMC6098007; doi:10.1038/s41598-018-29658-2)
Supplement: Supplementary file 1 — Supplementary Figures and data [file 41598_2018_29658_MOESM1_ESM.pdf]

Supplementary Data File

Title:
TDP-25 ROUTING TO AUTOPHAGY AND PROTEASOME AMELIORATES ITS AGGREGATION IN AMYOTROPHIC LATERAL SCLEROSIS TARGET CELLS.

Authors:
***Maria Elena Cicardi* [1]*, Riccardo Cristofani* [1]*, Paola Rusmini* [1]*, Marco Meroni* [1]*, Veronica Ferrari*[1]*, Giulia Vezzoli*[1]*, Barbara Tedesco*[1]*, Margherita Piccolella* [1]*, Elio Messi* [1]*, Mariarita Galbiati*[1]*, Alessandra Boncoraglio*[1]*, Serena Carra* [2]*, Valeria Crippa*[1]*, Angelo Poletti* [1,3§].**

Afflation
[1] Department of Excellence: Dipartimento di Scienze Farmacologiche e Biomolecolari (DiSFeB), Centro di Eccellenza sulle Malattie Neurodegenerative, Università degli Studi di Milano, Milano, (Italy).
[2] Dipartimento di Scienze Biomediche, Metaboliche e Neuroscienze, Università degli Studi di Modena e Reggio Emilia, and Center for Neuroscience and Neurotechnology, Modena, Italy
[3] Centro InterUniversitario sulle Malattie Neurodegenerative, Università degli Studi di Firenze, Genova, Roma Tor Vergata and Milano, Italy

[§] Corresponding authors: Angelo Poletti. Dipartimento di Scienze Farmacologiche e Biomolecolari (DiSFeB), Università degli Studi di Milano - Via Balzaretti 9, 20133 Milano (Italy). ph. +39-02-50318215; fax: +39-02-50318204_ e-mail: <angelo.poletti@unimi.it>

FIGURE S1

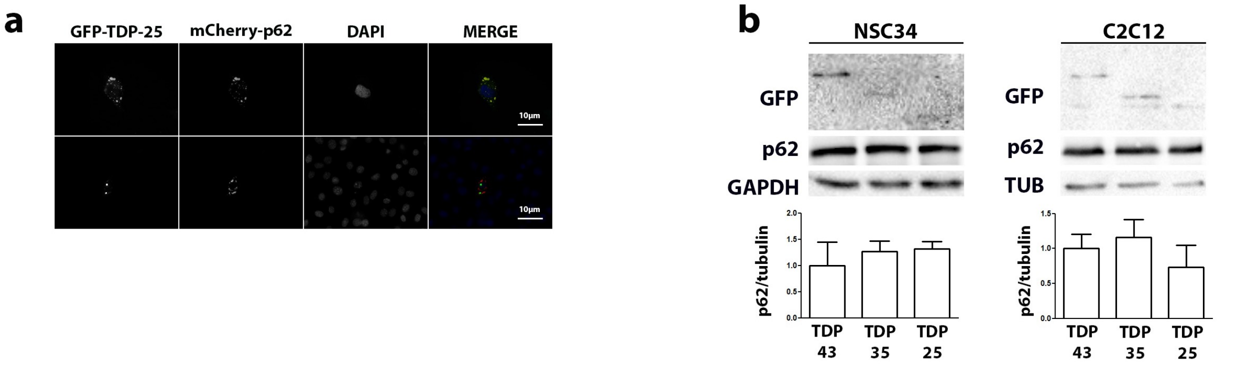

Figure S1: p62 localization and levels upon GFP-TDP-25 expression.
(a) Microscopy analysis. NSC34 and C2C12 were transfected with GFP-TDP-25 and mCherry-p62. Green: GFP-TDP-25. Red: mCherry-p62. Nuclei staining: DAPI. Image were acquired with 63X magnification.
(b) NSC34 and C2C12 were transfected with GFP-TDP-43, GFP-TDP35 and GFP-TDP-25. WB shows GFP-TDPs protein levels and p62 protein levels. Tubulin/GAPDH were used as loading control. Graphics show p62 levels quantification normalized on tubulin.

FIGURE S2

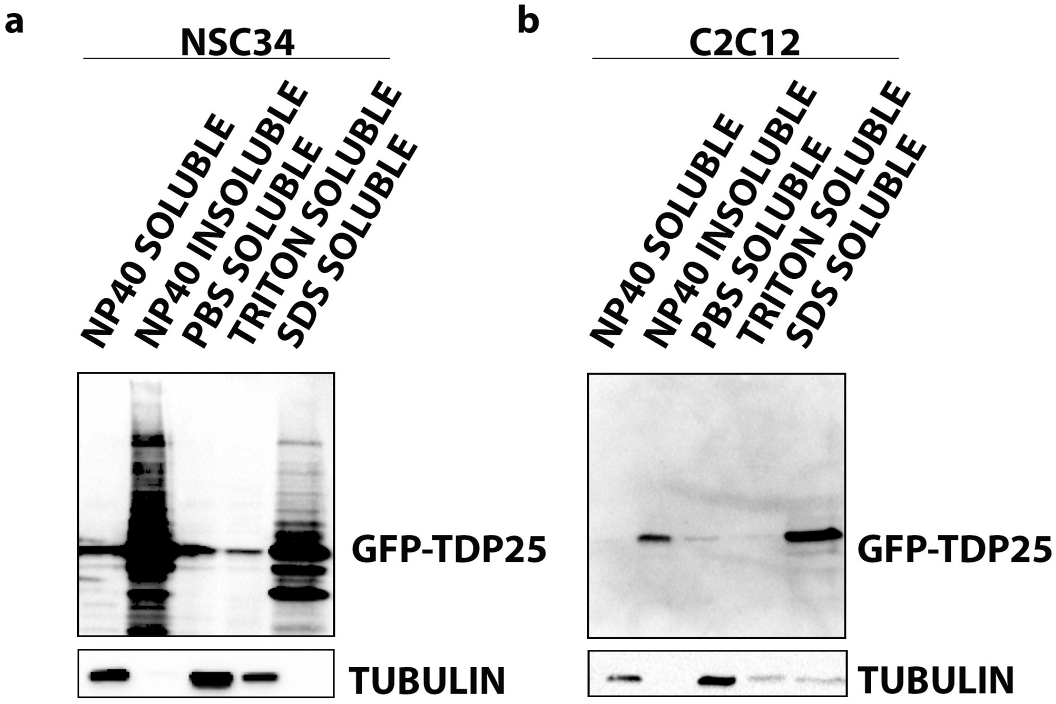

Figure S2: Comparison between fractionation and NP-40 extraction.
(a) Western Blot shows both extracts of fractionation and extracts from NP-40 extraction of NSC34 transiently overexpressing GFP-TDP-25.
(b) Western Blot shows both extracts of fractionation and extracts from NP-40 extraction of C2C12 transiently overexpressing GFP-TDP-25.

FIGURE S3

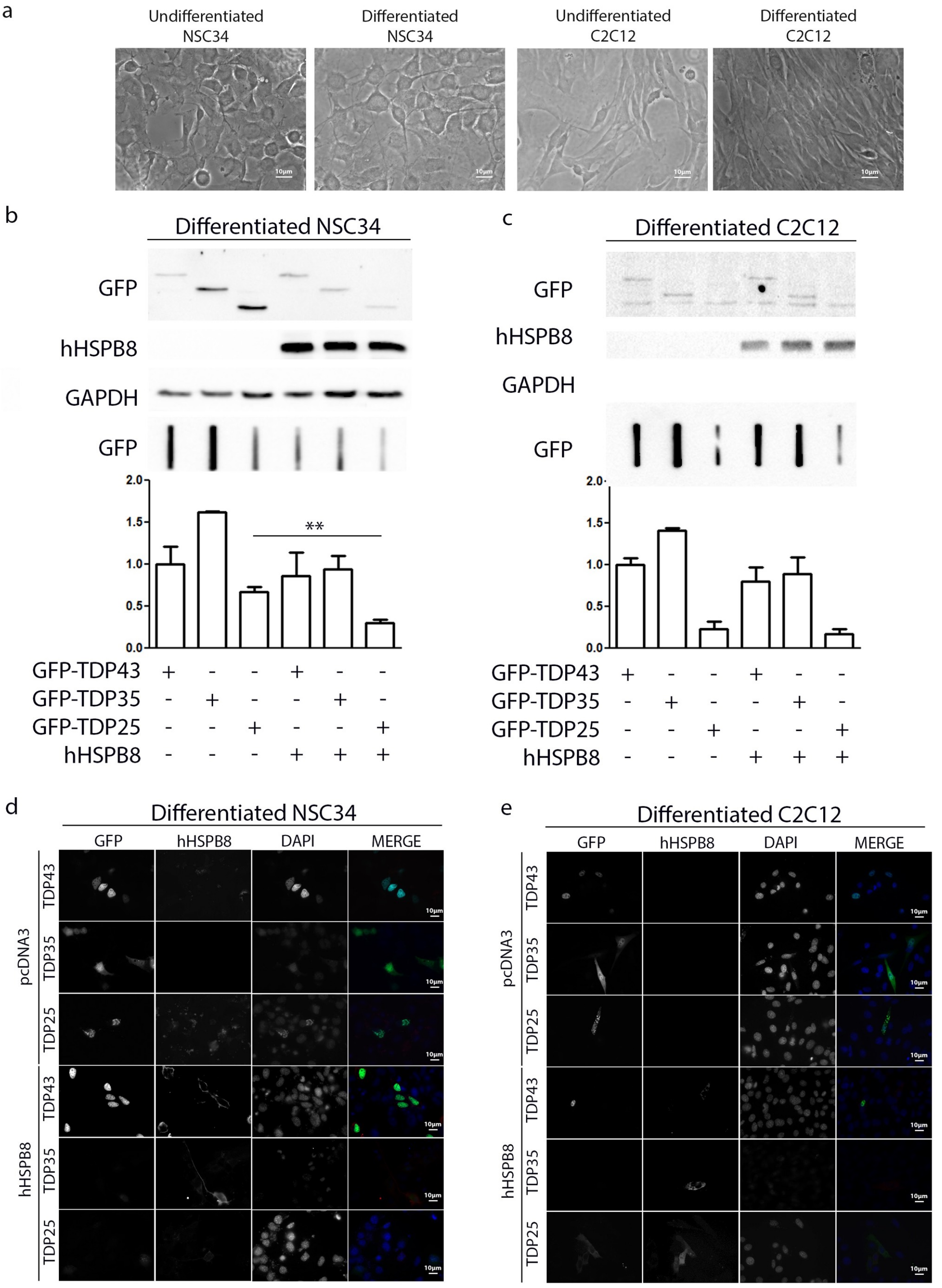

Figure S3: HspB8 pro-degradative activity in differentiated NSC34 and C2C12.
(a) bright field acquisition of undifferentiated and differentiated NSC34 and C2C12 cells. 32X magnification.
(b) Microscope analysis. Differentiated NSC34 transiently overexpressing GFP-TDP-43, GFP-TDP-35 and GFP-TDP-25, and co-transfected with pCI-HspB8 or pcDNA3 as control vector. 63X magnification. Green: GFP-TDPs; Red: hHspB8; nuclei staining: DAPI.
(c) Microscope analysis. Differentiated C2C12 transiently overexpressing GFP-TDP-43, GFP-TDP-35 and GFP-TDP-25, and co-transfected with pCI-HspB8 or pcDNA3 as control vector. 63X magnification. Green: GFP-TDPs; Red: hHspB8; nuclei staining: DAPI.
(d) Differentiated NSC34 transiently overexpressing GFP-TDP-43, GFP-TDP-35 and GFP-TDP-25. Upper inset shows PBS extracts WB analysis. Middle inset shows PBS extracts FRA analysis. Lower inset shows quantification of FRA analysis (* p<0,05). (e) Differentiated C2C12 transiently overexpressing GFP-TDP-43, GFP-TDP-35 and GFP-TDP-25.
Upper inset shows PBS extracts WB analysis. Middle inset shows PBS extracts FRA analysis. Lower inset shows quantification of FRA analysis.

FIGURE S4

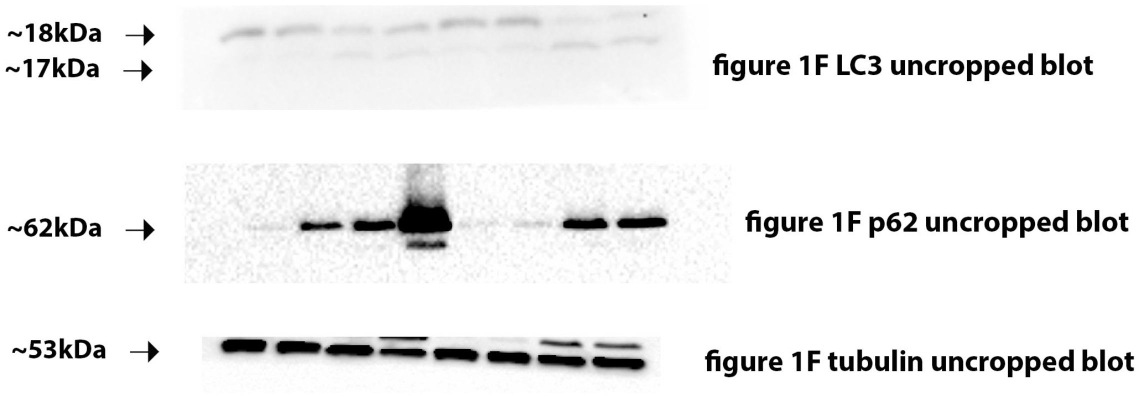

Figure S4: Uncropped Western Blot from Figure 1F.
Blots were halved in order to process the membrane against different antibodies, so the entire blots are not available.

FIGURE S5

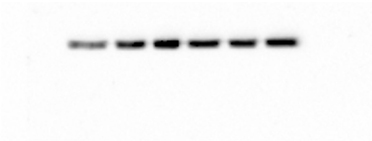

figure 2A tubulin corresponding to PBS fraction

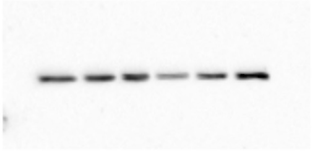

figure 2A tubulin corresponding to TRITON fraction

figure 2A tubulin corresponding to SDS fraction

figure 2A tubulin corresponding to FA fraction

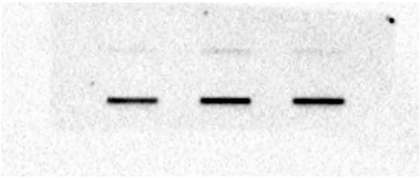

Uncropped FRA fig 2C antibody: FLAG

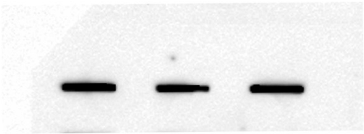

Uncropped FRA fig 2C antibody: GFP

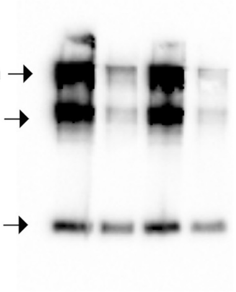

Uncropped WB fig 2D antibody: FLAG

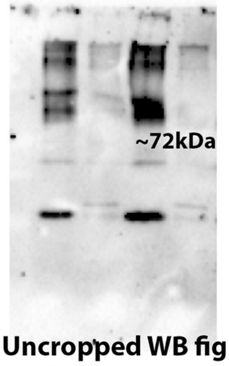

Uncropped WB fig 2D antibody: TDP

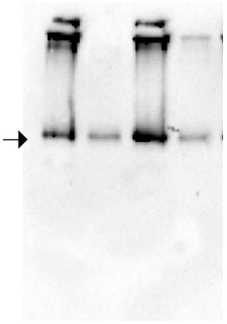

Uncropped WB fig 2D antibody: GFP

Figure S5: Uncropped Western Blot and Filter Retardation Assay from Figure 2 A, C, D.
Blots were halved in order to process the membrane against different antibodies, so the entire blots are not available.

FIGURE S6

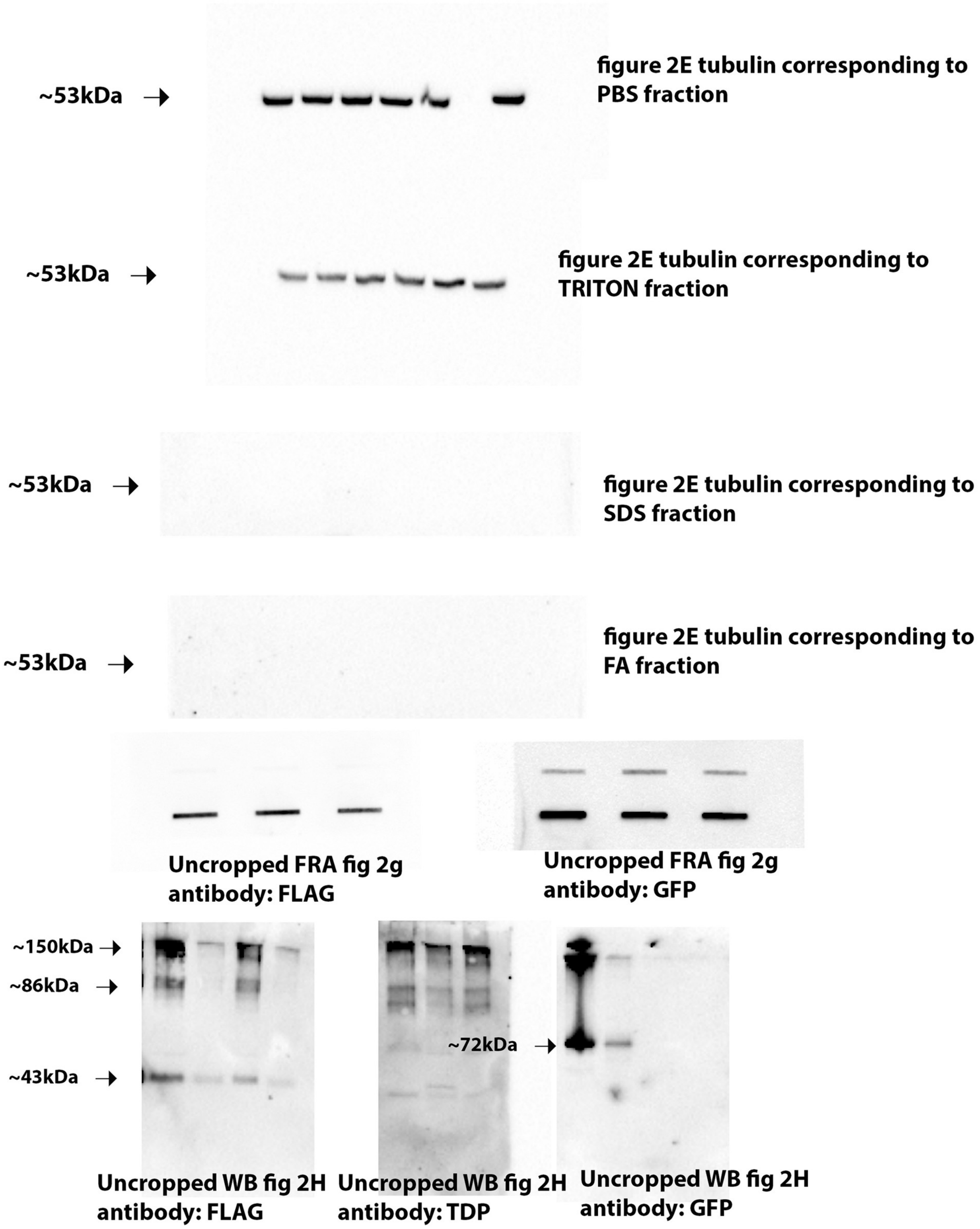

Figure S6: Uncropped Western Blot and Filter Retardation Assay from Figure 2 E, G, H.
Blots were halved in order to process the membrane against different antibodies, so the entire blots are not available.

FIGURE S7

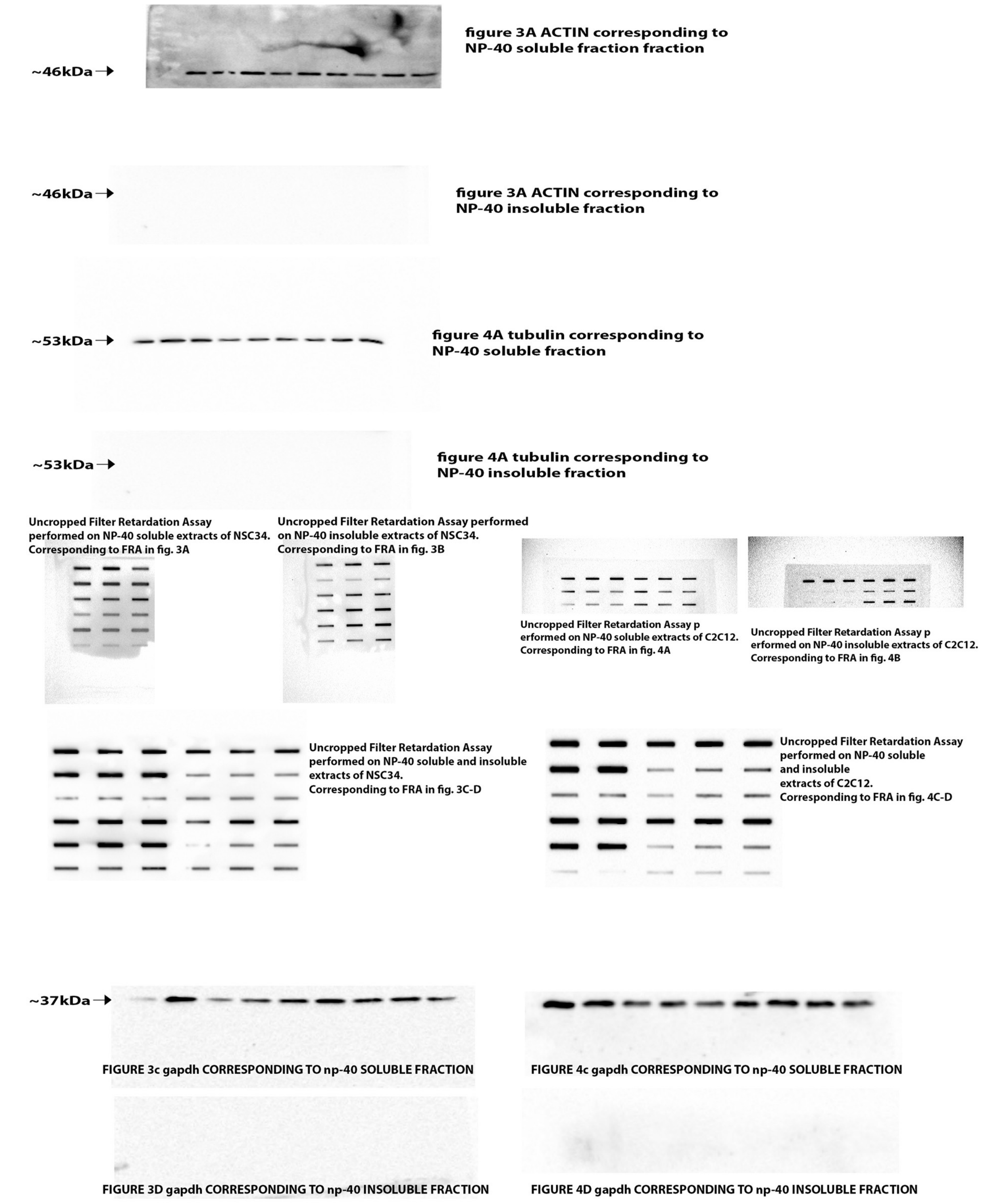

Figure S7: Uncropped Western Blot and Filter Retardation Assay from Figure 3 and Figure 4.
Blots were halved in order to process the membrane against different antibodies, so the entire blots are not available.

FIGURE S8

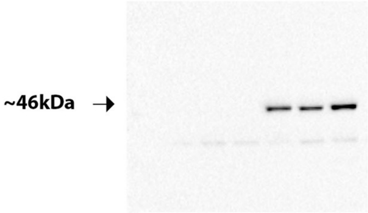

figure 5A HA antibody corresponding to Bag1 in NP-40 soluble fraction

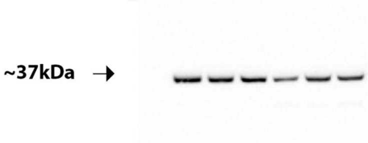

figure 5A GAPDH corresponding to NP-40 soluble fraction fraction

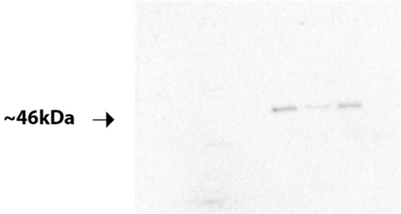

figure 5A HA antibody corresponding to Bag1 in NP-40 insoluble fraction

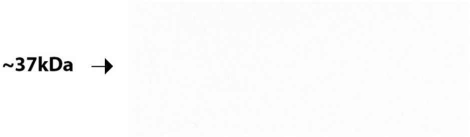

figure 5A GAPDH corresponding to NP-40 insoluble fraction fraction

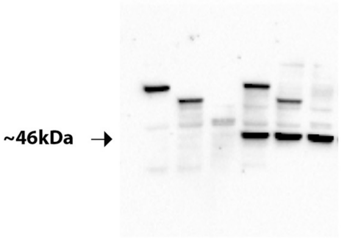

figure 5C HA antibody corresponding to Bag1 in NP-40 soluble fraction

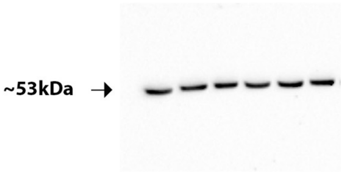

figure 5C tubulin corresponding to NP-40 soluble fraction fraction

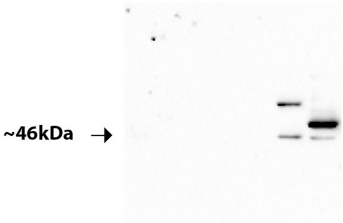

figure 5C HA antibody corresponding to Bag1 in NP-40 insoluble fraction

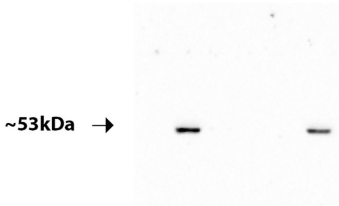

figure 5C tubulin corresponding to NP-40 insoluble fraction fraction

Figure S8: Uncropped Western Blot from Figure 5.
Blots were halved in order to process the membrane against different antibodies, so the entire blots are not available.

FIGURE S9

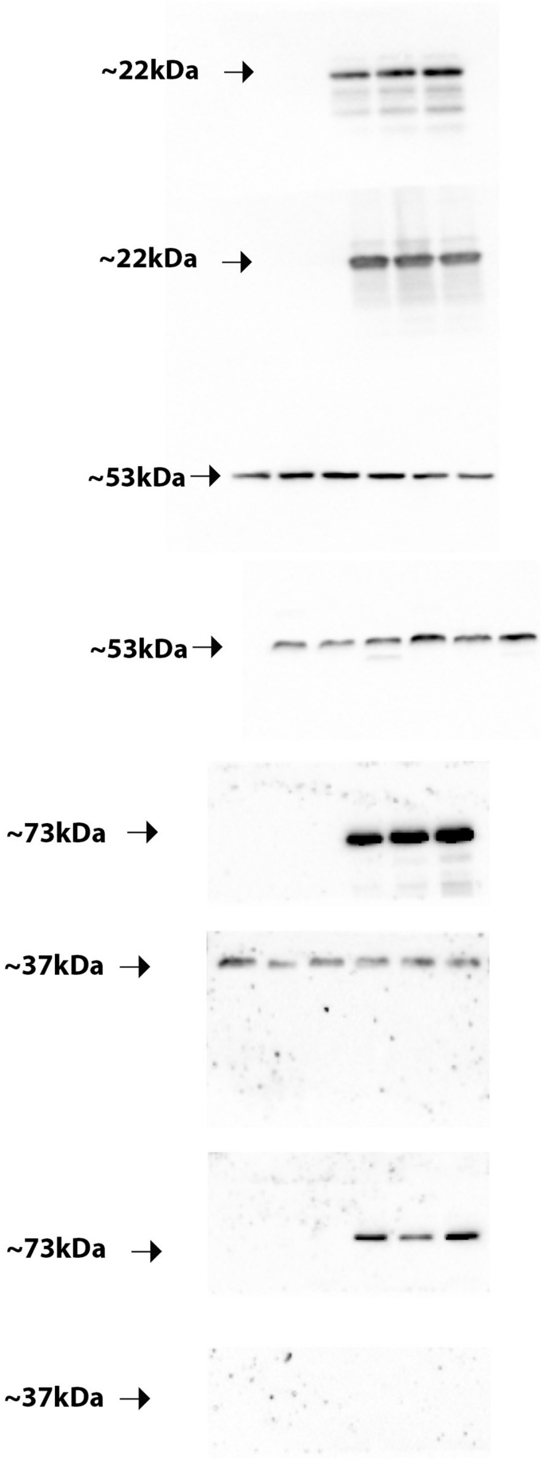

figure 6A HspB8 corresponding to NP-40 soluble fraction

figure 6A HspB8 corresponding to NP-40 insoluble fraction

figure 6A tubulin corresponding to NP-40 soluble fraction

figure 6A tubulin corresponding to NP-40 insoluble fraction

figure 6C Bag3 corresponding to NP-40 soluble fraction

figure 6C GAPDH corresponding to NP-40 soluble fraction

figure 6C Bag3 corresponding to NP-40 insoluble fraction

figure 6C GAPDH corresponding to NP-40 insoluble fraction

Figure S9: Uncropped Western Blot from Figure 6.
Blots were halved in order to process the membrane against different antibodies, so the entire blots are not available.

FIGURE S10

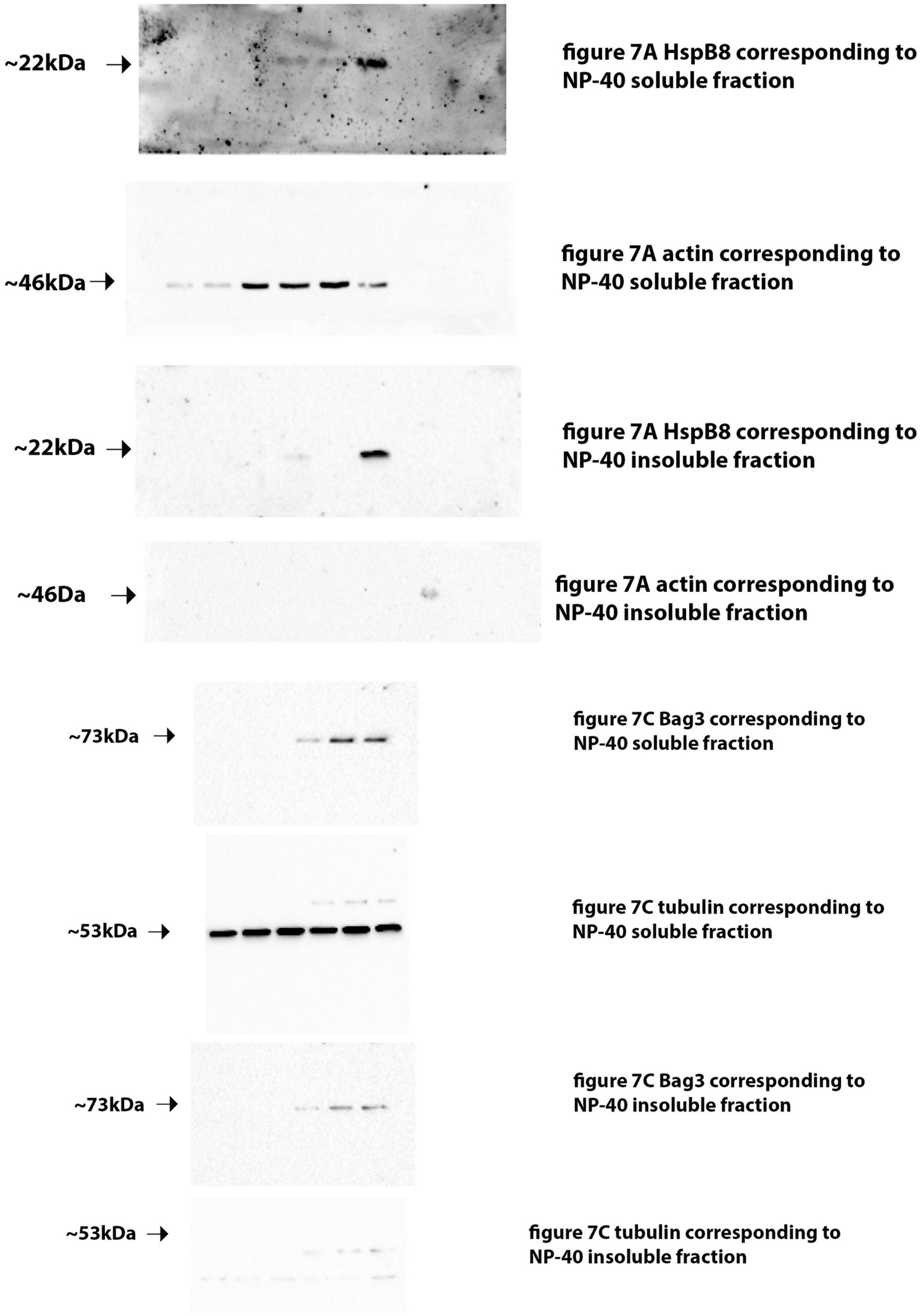

Figure S10: Uncropped Western Blot from Figure 7.
Blots were halved in order to process the membrane against different antibodies, so the entire blots are not available.
